# Effects of Mindfulness and Exercise on Growth Factors, Inflammation, and Stress Markers in Chronic Stroke: The MindFit Project Randomized Clinical Trial

**DOI:** 10.3390/jcm14082580

**Published:** 2025-04-09

**Authors:** Adrià Bermudo-Gallaguet, Mar Ariza, Daniela Agudelo, Neus Camins-Vila, Maria Boldó, Sarah Peters, Angelika Katarzyna Sawicka, Rosalia Dacosta-Aguayo, Juan José Soriano-Raya, Marc Via, Imma C. Clemente, Alberto García-Molina, Maria José Durà Mata, Pere Torán-Monserrat, Kirk I. Erickson, Maria Mataró

**Affiliations:** 1 Departament de Psicologia Clínica i Psicobiologia, Facultat de Psicologia, Universitat de Barcelona (UB), Passeig de la Vall d’Hebron, 171, 08035 Barcelona, Spain; abermudo@ub.edu (A.B.-G.); mariza@ub.edu (M.A.); daniela.agudelo@ub.edu (D.A.); sarahpppeters@gmail.com (S.P.); juan.jose.soriano@ub.edu (J.J.S.-R.); mvia@ub.edu (M.V.); iclemente@ub.edu (I.C.C.); 2 Institut de Neurociències, Universitat de Barcelona, Passeig de la Vall d’Hebron, 171, 08035 Barcelona, Spain; 3 Institut de Recerca Sant Joan de Déu Santa Rosa 39-57, 08950 Esplugues de Llobregat, Spain; 4 Institut Nacional d’Educació Física de Catalunya (INEFC), Universitat de Barcelona (UB), 08038 Barcelona, Spain; ncaminvi16@alumnes.ub.edu; 5 Servei de Rehabilitació, Hospital Universitari Germans Trias i Pujol, Campus Can Ruti, 08916 Badalona, Spain; mboldo.ics@gencat.cat (M.B.); mjdura.germanstrias@gencat.cat (M.J.D.M.); 6 Institut de Recerca Germans Trias i Pujol (IGTP), Campus Can Ruti, 08916 Badalona, Spain; rdacostaa@gmail.com (R.D.-A.); agarciam@guttmann.com (A.G.-M.); ptoran.bnm.ics@gencat.cat (P.T.-M.); 7 Applied Cognitive Neuroscience Lab, Department of Neurophysiology, Neuropsychology and Neuroinformatics, Medical University of Gdansk, 80-210 Gdansk, Poland; ange.sawicka@gmail.com; 8 Unitat de Suport a la Recerca Metropolitana Nord, Fundació Institut Universitari per a la Recerca a l’Atenció Primària de Salut Jordi Gol i Gurina, 08303 Mataró, Spain; 9 Institut Guttmann, Universitat Autònoma de Barcelona, 08916 Badalona, Spain; 10 Grup de REcerca Multidisciplinar en Salut i Societat (GREMSAS), 08303 Mataró, Spain; 11 Department of Medicine, Faculty of Medicine, Universitat de Girona, 17003 Girona, Spain; 12 Advent Health Research Institute, Neuroscience, Orlando, FL 32803, USA; kirk.erickson@adventhealth.com; 13 Department of Psychology, University of Pittsburgh, Pittsburgh, PA 15260, USA

**Keywords:** stroke, mindfulness, physical exercise, computerized cognitive training, neuroplasticity, growth factors, inflammation, stress

## Abstract

**Background/Objectives**: Stroke often leads to persistent cognitive and emotional impairments, which rehabilitation may mitigate. However, the biological mechanisms underlying such improvements remain unclear. This study investigated whether supplementing computerized cognitive training (CCT) with mindfulness-based stress reduction (MBSR) or physical exercise (PE) modulated biomarkers of neuroplasticity, inflammation, and stress in patients with chronic stroke compared to CCT alone. We also explored whether biomarker changes mediated or correlated with behavioral improvements. **Methods**: In a three-arm, single-blind, randomized controlled trial (NCT04759950), 141 patients with chronic stroke were randomized (1:1:1) to MBSR+CCT, PE+CCT, or CCT-only for 12 weeks. Plasma levels of brain-derived neurotrophic factor (BDNF), insulin-like growth factor-1 (IGF-1), vascular endothelial growth factor (VEGF), C-reactive protein (CRP), interleukin-6 (IL-6), and cortisol were measured at baseline and post-intervention. Cognitive, mental health, mindfulness, and fitness outcomes were also assessed. Between- and within-group changes were analyzed using ANCOVA and paired *t*-tests. Per-protocol and complete-case analyses were conducted. **Results**: Among the 109 participants with ≥80% adherence, the only significant between-group difference was for VEGF: it remained stable in the MBSR+CCT group but declined in PE+CCT and CCT-only. Within-group analyses showed significant decreases in cortisol in MBSR+CCT and PE+CCT, while IGF-1 levels declined across all groups. In contrast, BDNF, IL-6, and CRP did not show significant changes, and biomarker changes were not significantly associated with behavioral improvements. Complete-case analysis (*n* = 126) yielded similar findings. **Conclusions**: Our findings suggest that combining MBSR or PE with CCT may modulate certain biological processes relevant to stroke recovery. MBSR may help maintain VEGF levels, which could support vascular health, while MBSR and PE may contribute to lowering cortisol levels. However, since no clear association with behavioral improvements was found, further research is needed to determine the clinical relevance of these biomarker changes in stroke recovery.

## 1. Introduction

Stroke remains a leading cause of death and long-term disability worldwide, with almost 12 million new cases annually [1]. According to the most recent estimates from the Global Burden of Disease Study and the World Stroke Organization, stroke is currently the second-leading cause of death and the third-leading cause of disability globally [1,2]. The number of people affected by stroke is expected to increase in the coming decades due to population aging and increased exposure to vascular risk factors [3]. Although survival rates have improved thanks to advances in acute treatments [4], many stroke survivors experience chronic disabilities, including physical impairments, cognitive deficits, depression, behavioral changes, and complications such as dysphagia and malnutrition, all of which can significantly reduce quality of life [5,6,7,8,9,10]. Furthermore, stroke is a major risk factor for dementia, even in patients with mild or transient cognitive impairment [6].

Cognitive rehabilitation is the gold standard for addressing post-stroke cognitive deficits [11]. Within this context, computerized cognitive training (CCT) has emerged as a prominent digital alternative to traditional methods. Its accessibility and ease of at-home implementation, along with its ability to provide individualized cognitive exercises, make it a valuable complement to existing approaches [12]. Meta-analyses suggest that CCT enhances cognitive function in stroke survivors, potentially by leveraging mechanisms of neuroplasticity [13,14,15]. Regular engagement in repetitive and structured cognitive tasks may activate specific neural circuits simultaneously, reinforcing synaptic connections and promoting adaptive changes in brain function [16,17]. In stroke populations, some evidence suggests that CCT may enhance hippocampal functional connectivity with other brain regions [18,19], although a study failed to detect structural connectivity changes [20]. A key hypothesized pathway involves the upregulation of brain-derived neurotrophic factor (BDNF) [21], a neurotrophin that is abundantly expressed in the cerebral cortex and hippocampus, playing a critical role in synaptic plasticity, neuronal development, and neuroprotection [22]. While increases in BDNF following cognitive training have been demonstrated in other populations (e.g., [23,24]), evidence in stroke survivors remains scarce, with studies to date reporting predominantly negative findings [25]. Despite these findings, the molecular mechanisms underlying CCT’s benefits in stroke populations remain poorly understood and warrant further investigation [25].

Emerging evidence indicates that combining rehabilitation methods can enhance neuroplasticity and brain health, thereby improving stroke recovery across multiple domains. For instance, studies on animal models with stroke demonstrate that exercise programs combined with enriched environments enhance brain, cognitive, and physical functions [26,27]. Likewise, environmental enrichment and multimodal interventions tailored for patients with stroke seek to engage them in simultaneous physical, sensory, cognitive, emotional, and social activities [28,29]. Although research on these interventions is still in its early stages, they show promise for improving recovery in early rehabilitation and potentially preserving brain health or preventing cognitive decline and dementia during chronic phases [28,30]. Among various approaches, mindfulness-based interventions (MBIs) and physical exercise (PE) programs have emerged as probably effective strategies to incorporate in post-stroke rehabilitation [31,32,33,34,35,36,37]. These interventions may complement cognitive training by enhancing cognitive function, mental health, and sensorimotor skills through distinct yet potentially overlapping physiological mechanisms (e.g., see [38]).

MBIs are thought to promote psychological and physical health by modulating the body’s stress-response systems, including reducing the reactivity of the sympathetic nervous system (e.g., lowering catecholamines and blood pressure) and the hypothalamic–pituitary–adrenal (HPA) axis [39]. The HPA axis regulates cortisol release, which supports energy allocation and immune responses during acute stress [40]. Chronic stress, however, disrupts this balance, resulting in prolonged HPA activation, elevated cortisol levels, glucocorticoid resistance, and systemic inflammation [41]. According to systematic reviews and meta-analyses in healthy and clinical non-stroke populations, MBIs may lower cortisol levels, reduce inflammation markers such as C-reactive protein (CRP) and interleukin-6 (IL-6), and enhance immune function, including increased CD4+ T lymphocytes and delayed immune cell aging [42,43,44,45,46,47,48,49,50,51]. One meta-analysis further links MBIs to increased peripheral BDNF levels [52]. This effect may result, in part, from the reduction in systemic inflammation, which is known to interfere with several BDNF-signaling pathways [53]. Despite these findings, which offer preliminary evidence of the molecular effects of MBIs in various populations, there is, to our knowledge, a lack of studies directly examining these effects in stroke.

PE supports cognitive, emotional, and brain health through various interrelated biological mechanisms, many of which remain under active investigation [54,55,56,57]. Preclinical studies consistently show that PE enhances neuroplasticity and hippocampal neurogenesis, primarily via the upregulation of mature BDNF expression [58]. This effect is synergistically supported by increased levels of other growth factors, such as insulin-like growth factor-1 (IGF-1) and vascular endothelial growth factor (VEGF), which collectively promote neurogenesis, angiogenesis, and synaptic remodeling [54,55,57]. In stroke populations, acute aerobic exercise reliably elevates circulating neurotrophin levels; however, the outcomes of regular PE programs remain variable [59]. While some trials report significant increases in BDNF and IGF-1 [60,61,62], others do not observe changes in these or VEGF [25,63,64], likely due to heterogeneity in PE protocols and sample characteristics. Notably, a recent meta-analysis identified exercise intensity as a critical variable, highlighting high-intensity aerobic exercise as particularly effective in elevating BDNF levels among patients with stroke [65]. PE may also exert anti-inflammatory and antioxidant effects and modulate the HPA axis [54,55,57]. Nevertheless, evidence for reductions in pro-inflammatory cytokines, oxidative stress markers, and cortisol levels is limited and inconsistent in stroke populations [63,66,67,68]. Furthermore, there is a gap in the research on resistance training or combined aerobic-strength programs, even though one meta-analysis showed that these combined approaches may provide the most cognitive benefits for stroke survivors [36].

Given the overlapping biological mechanisms of CCT, MBIs, and PE, combining these interventions could plausibly enhance their molecular effects, leading to greater impacts on cognition and emotion. Nonetheless, this is a relatively new area of study in stroke. Some studies have looked at interventions that combine PE with cognitive training, and their results suggest that these combinations may yield superior cognitive benefits compared to individual therapies [69]. However, few have explored associated molecular changes. As an exception, one trial did not observe increases in BDNF or IGF-1 after a 10-week aerobic plus CCT intervention [25]. Regarding MBIs, no studies to date have examined their combination with cognitive training in terms of behavioral or molecular outcomes in patients with stroke.

The MindFit Project was a randomized controlled trial designed to test whether integrating mindfulness-based stress reduction (MBSR) or PE with CCT could yield greater cognitive and emotional improvements compared to CCT alone [70]. In a prior study from the same project, currently under review, changes in primary cognitive and mental health outcomes did not significantly differ between groups. Pre–post analyses showed cognitive improvements across all groups, with the greatest mental health benefits observed in the MBSR+CCT group. Secondary analyses found that the PE+CCT group showed significantly greater improvements on specific cognitive tests and significantly larger gains in physical fitness. MBSR+CCT also enhanced mindfulness, which significantly mediated reductions in depressive symptoms in that group [71]. Qualitative insights from a substudy further supported these findings, providing a deeper understanding of the unique benefits of each intervention and highlighting their multifaceted impact [72]. While these findings highlight promising effects on specific outcomes, understanding the underlying molecular changes remains a critical next step.

This study investigates the molecular mechanisms and behavioral correlates of the three intervention groups in the MindFit Project—MBSR+CCT, PE+CCT, and CCT-only. Specifically, we assess (i) between- and within-group changes in biomarkers related to neuroplasticity (BDNF, IGF-1, VEGF), inflammation (CRP, IL-6), and stress (cortisol) and (ii) their associations with cognitive, mental health, mindfulness, and fitness outcomes. We hypothesize that adding MBSR or PE to CCT will lead to additional—and potentially distinct—biomarker changes compared to CCT alone, given the different mechanisms by which mindfulness and exercise may influence biological processes. Furthermore, we expect these biomarker changes to be differentially associated with behavioral outcomes depending on the intervention arm. However, due to the limited and often mixed findings in chronic stroke populations, we do not propose specific a priori hypotheses for each biomarker. Instead, we adopt an exploratory approach to identify emergent patterns of change and their relationships with behavioral outcomes.

## 2. Materials and Methods

### 2.1. Design and Setting

The MindFit Project was a single-center, parallel, three-arm, single-blind randomized controlled trial conducted by the Faculty of Psychology at the Universitat de Barcelona in collaboration with Hospital Germans Trias i Pujol and the Guttmann Institute. A total of 141 patients with chronic stroke were randomly assigned in a 1:1:1 ratio to one of three groups: MBSR+CCT, PE+CCT, or CCT-only. The interventions spanned 12 weeks, with evaluations occurring at baseline and post-intervention. Due to COVID-19 restrictions, all interventions and most assessments were conducted online. The study received approval from the Universitat de Barcelona Ethics Committee (IRB00003099) and the rest of the participating centers, and the trial protocol was registered in ClinicalTrials.gov (NCT04759950) and published [70]. Furthermore, this study follows the CONSORT guidelines [73], and a detailed CONSORT checklist is provided in the Supplementary Materials (Table S1).

### 2.2. Recruitment

Participants were recruited from Catalonia and other regions in Spain using various strategies. These included advertisements on social media, press releases in local media, and direct outreach to hospitals and patient associations. The recruitment period lasted from June 2020 to February 2021.

### 2.3. Eligibility Criteria

The inclusion and exclusion criteria pre-specified in our protocol are summarized in Table 1. However, delays due to the COVID-19 pandemic and the gap between recruitment and randomization led to some initially eligible participants no longer meeting the criteria for age and time since stroke. To address these unforeseen delays, the criteria were modified to include two participants aged over 80 and 11 participants whose strokes occurred more than 60 months earlier.

### 2.4. Randomization and Blinding

Participants were assigned to one of three groups using a simple randomization process with equal allocation (1:1:1). The allocation sequence was generated by an independent biostatistician using random-sequence-generation software and stored in a password-protected database accessible exclusively to the randomization staff not involved in any other process. Allocation was concealed through a centralized procedure, and group assignments were revealed only after enrollment and baseline assessments were completed. This method ensured that neither the recruiting personnel nor the outcome assessors could predict or influence the allocation sequence.

After randomization, participants and intervention providers were necessarily aware of group allocations due to the different nature of the interventions. In contrast, outcome assessors and data analysts remained blinded to them to ensure unbiased data collection and interpretation. To maintain blinding, outcome assessors had access only to the materials necessary for administering and scoring assessments. They were not provided with details about the intervention and had no contact with intervention providers. Participants were identified using generic IDs to prevent assessors from inferring group allocation and were instructed not to disclose their assignments. Additionally, data analysts worked with de-identified datasets, and group allocation was unmasked only after all analyses were completed.

### 2.5. Interventions

#### 2.5.1. General Overview

Participants were allocated to one of three intervention groups: (1) MBSR+CCT, (2) PE+CCT, and (3) CCT-only. These interventions were conducted remotely, five days a week, over 12 weeks. Participants in the combined groups were free to choose the order in which they completed the MBSR or PE and CCT components. The total intervention duration for the combined groups was twice that of the active control group (CCT-only), reflecting the additional components of MBSR or PE.

MBSR and PE interventions included weekly synchronous sessions through the professional version of Zoom, with group sizes ranging from 8 to 12 participants, in addition to other autonomous sessions. Conversely, CCT was provided on an individual basis. All participants were encouraged to share their experiences and interact via WhatsApp groups specific to their intervention arm, including those in the CCT-only group, which lacked synchronous sessions. This approach aimed to mitigate the potential confounding effects of social support.

Below is a brief description of each intervention. The study protocol provides detailed information on the materials, accommodations, specific exercises, and workload progression.

#### 2.5.2. Mindfulness-Based Stress Reduction

The MBSR program, facilitated by a certified mindfulness instructor, followed Jon Kabat-Zinn’s foundational protocol [76] with slight modifications to expand the traditional 8-week structure to 12 weeks (Table 2). Core components included guided meditation techniques (e.g., body scan, breathing exercises), gentle Hatha yoga, and mindfulness-based practices for daily life. Weekly activities included a 150-min group session conducted online via Zoom and four self-guided daily practices (20–40 min per session).

#### 2.5.3. Physical Exercise

The PE intervention program was based on the American Stroke Association’s exercise recommendations for stroke survivors [77]. It was a multicomponent program that combined aerobic and strength training (Table 3). Participants attended three weekly group videoconference sessions led by a physiotherapist and a strength and conditioning specialist. Two sessions, lasting 60 min each, concentrated on strength, agility, and balance, while the third, lasting 45 min, focused on aerobic conditioning. In addition to the group sessions, participants completed two independent walking sessions per week, each consisting of 45 min of moderate-intensity exercise.

#### 2.5.4. Computerized Cognitive Training

The CCT intervention utilized the Guttmann NeuroPersonalTrainer^®^ (GNPT^®^) software (version 2.13.2) (Fundació Institut Guttmann, Badalona, Spain), a computerized telemedicine tool for personalized cognitive rehabilitation [12]. Participants completed five 45-min sessions weekly at home. Each session included tasks targeting executive function, memory, and attention. The software dynamically adjusted task difficulty based on each participant’s baseline cognitive abilities and real-time performance to ensure an appropriate level of challenge and engagement.

### 2.6. Intervention Adherence and Safety

Intervention adherence was monitored through participant attendance during supervised sessions and detailed diaries filled by the participants documenting autonomous sessions. Adherence to PE and CCT was calculated as the proportion of completed to planned sessions (each session lasted approximately the same amount of time: 45–60 min). In contrast, for MBSR, which included variable session lengths (20–40 min of independent practice and 250 min of group sessions), adherence was measured as the total time participants engaged relative to the planned program duration. Overall adherence was calculated as the average adherence to the individual components for participants in combined-intervention groups.

Safety monitoring was conducted throughout the study. Participants were encouraged to report any adverse events in their daily logs or during weekly check-ins with intervention facilitators. Reported adverse events were documented and evaluated.

### 2.7. Assessments and Outcome Measures

Changes in blood biomarkers are the main outcomes of this study. Additionally, we used the data from behavioral outcomes to assess potential correlations with biomarker changes or to adjust for confounding effects. Outcome measures were collected within 15 days before the start and within 15 days after the end of the intervention period.

#### 2.7.1. Baseline Measures

Baseline demographic and clinical data were collected during initial assessments. A neuropsychologist gathered demographic details, such as age, sex, ethnicity, and years of education, during the screening interview. The same specialist also administered the Mini-Mental State Examination (MMSE) to evaluate the global cognitive status [75]. Concurrently, a medical physician documented stroke characteristics (date, type, affected artery, side) and other medical information (additional diagnoses and medications). This physician also assessed neurological functionality and disability using the National Institutes of Health Stroke Scale (NIHSS), which evaluates stroke severity [74]; the Barthel Index, which measures independence in daily activities [80]; and the modified Rankin Scale (mRS), which assesses overall disability [81].

#### 2.7.2. Biomarker Outcomes

Blood samples were collected in the Germans Trias i Pujol Hospital. Samples were drawn between 08:00 and 09:00 a.m., and all patients received instructions not to eat or drink anything other than water 8 h before. Blood samples collected in EDTA vacutainer tubes were processed for plasma or frozen at −20 °C. Samples were analyzed by CEREMET, the Metabolism Research Center at the University of Barcelona (http://www.ub.edu/ceremet/, accessed on 24 February 2025), in compliance with ISO 9001:2015 standards [82]. BDNF, IGF-1, VEGF, CRP, IL-6, and cortisol were all analyzed in plasma. Plasma that had a low platelet count was separated by double centrifugation. Aliquots were frozen at −80 °C for further analysis. To measure the values of BDNF, IGF-1, VEGF, IL-6, and cortisol, ELISA kits from R&D Systems (Minneapolis, MN, USA) were used. A turbidimetric method using reagents from RAL (Sant Joan Despí, Spain) was used to obtain CRP levels in plasma. All experiments were conducted following the guidelines provided by the kits’ manufacturers. Although stromal cell-derived factor 1-alpha (SDF1-α), nerve growth factor (NGF), and homocysteine were initially listed as biomarker outcomes on ClinicalTrials.gov, they were not analyzed due to the high associated costs. The finalized study protocol specified the biomarkers that were ultimately measured and reported.

#### 2.7.3. Behavioral Outcomes

Behavioral outcomes included cognitive, mental health, mindfulness, physical fitness, and lifestyle measures.

Cognitive outcomes were assessed using a battery of standardized tests administered via the business version of Zoom by a licensed neuropsychologist. These included the Boston Naming Test-15 [83], Rey Auditory Verbal Learning Test [84], Rey-Osterrieth Complex Figure [85], Stroop Color and Word Test [86], Trail Making Test A and B [87], Verbal Fluency Test (letter and category fluency) [88], and selected subtests from the Wechsler Adult Intelligence Scale-III [89], including Digits, Digit Symbol Coding, Matrix, and Vocabulary. From this comprehensive battery of cognitive tests, we applied exploratory factor analysis with principal axis factoring and direct oblimin rotation [71]. This analysis identified three oblique factors, which explained approximately 68.72% of the total variance and were labeled Verbal Executive Functioning, Verbal Memory, and Visual Executive Functioning. Factor loadings and information about sampling adequacy are provided in the Supplementary Materials, Table S2.

Mental health was assessed using two widely validated tools: the Beck Depression Inventory-II (BDI-II) [90] and the Depression, Anxiety, and Stress Scales-21 (DASS-21) [91]. The BDI-II focuses on evaluating the severity of depressive symptoms. In contrast, the DASS-21 provides specific scores for depression, anxiety, and stress, along with a composite score representing the sum of these dimensions.

Mindfulness was assessed using two self-report questionnaires: the Mindful Attention Awareness Scale (MAAS), which measures awareness of the present moment [92], and the Five Facet Mindfulness Questionnaire (FFMQ), which evaluates mindfulness across five dimensions: observing, describing, acting with awareness, non-reactivity, and non-judging [93]. These dimensions were combined into an overall mindfulness score.

Physical fitness was assessed remotely using an adapted version of the Senior Fitness Test (SFT) [94]. Among the six standardized measures, this study focused on the three most relevant to the PE intervention: lower-body strength, upper-body strength, and aerobic capacity. Lower-body strength was measured using the 30 s Chair Stand Test (30CST), upper-body strength with the 30 s Arm Curl Test (30ACT), and aerobic capacity with the 2 min Step Test (2MST). All tests were conducted by a trained physiotherapist via a secure telemedicine platform, adhering to official SFT guidelines. Online functional assessments have been validated as safe, feasible, and comparable to in-person tests [95,96]. Anthropometric measurements were recorded to calculate body mass index (BMI). Additionally, the physiotherapist administered the Spanish short version of the Minnesota Leisure Time Physical Activity Questionnaire (VREM) to estimate energy expenditure in metabolic equivalent of task units [97].

Lifestyle measures, apart from the abovementioned VREM, included adherence to the Mediterranean diet, assessed with the Mediterranean Diet Adherence Screener (MedAS) [98], and sleep quality, evaluated using the Pittsburgh Sleep Quality Index (PSQI) [99]. All self-reported questionnaires, except for the VREM, were completed online via Microsoft Forms under a license from the University of Barcelona.

### 2.8. Statistical Analysis

Sample size calculation was based on expected effect sizes for primary cognitive and emotional outcomes reported in previous studies of mindfulness and exercise interventions in stroke populations. Using Granmo (v. 7.12), a sample size of 47 participants per group (three arms total) was calculated to achieve 80% power to detect a between-group difference of 0.75 standard deviations, assuming a common standard deviation of 1 and a two-tailed alpha of 0.05. This estimate accounted for an anticipated 20% dropout rate. Statistical analyses were performed using IBM SPSS Statistics for Windows (v. 29) and R (v. 4.4.1), with a significance level set at *p* < 0.05 (two-tailed).

We conducted a descriptive analysis to evaluate the distributions of variables, identify missing data, and detect outliers and censored data. BDNF, VEGF, IL-6, and CRP exhibited highly positively skewed distributions. Consequently, we applied a base-10 logarithmic transformation to reduce skewness and approximate normality at both time points. In contrast, the distributions of IGF-I and cortisol were approximately normal and did not require transformation. Figure S1 in the Supplementary Materials illustrates the distribution of raw and log-transformed baseline values.

For VEGF and CRP, 5.68% and 17.42% of values, respectively, were below the test’s detection limit. We substituted these non-detectable values with half of the test’s lower limit of quantification (LLOQ)—15.65 pg/mL for VEGF and 0.09 mg/L for CRP—a common approach in biomarker research [100]. This simple method preserves sample size and reduces bias compared to case-wise deletion. However, more advanced imputation techniques are available. Therefore, to enhance the robustness of our findings, we repeated the primary analyses for these biomarkers using a lognormal distribution-based imputation method, as described by Herbers et al. [100], and implemented it in the R package lnormimp [101]. This approach applies maximum likelihood estimation to fit a lognormal distribution to the observed data, after which non-detectable values are imputed by sampling from the fitted distribution below the LLOQ. The raw distributions of VEGF and CRP aligned with a lognormal model, supporting the suitability of this imputation technique. We generated five imputed datasets, applied our analysis pipeline to each, and pooled the results using Rubin’s rules with the mice [102] and miceadds [103] R packages.

We used analysis of covariance (ANCOVA) to assess intervention effects between groups. The dependent variable in each model was the biomarker’s difference score (post-intervention minus baseline). Covariates pre-specified in the study protocol included age, sex, years of education, time since stroke, and the baseline score of the outcome variable [70]. We verified ANCOVA assumptions using the R package performance [104], assessing linearity, normality of residuals, homogeneity of variances, influential cases, and collinearity. Log-transforming skewed biomarkers improved residual distributions and overall model performance. We performed post hoc pairwise comparisons with Bonferroni correction for significant between-group differences, ensuring stringent control of the family-wise error rate in these tests. Additionally, we analyzed within-group differences over time using paired *t*-tests. To address the multiple comparisons arising from assessing several biomarkers and tests, we utilized the false discovery rate (FDR) correction with the Benjamini–Hochberg method [105,106] for the ANCOVA *F*-tests and the paired *t*-tests. This approach, less restrictive than methods like Bonferroni correction, balances the control of Type I errors with maintaining statistical power [105].

To complement the primary ANCOVA analyses, we performed sensitivity analyses to account for potential additional confounders that might influence biomarker levels. Pearson partial correlations were used to assess relationships between a pool of variables and biomarker changes, adjusting for the predefined covariates and treatment groups. These candidate variables included stroke characteristics (number, type, and circulation of strokes), functional and disability scales (NIHSS, mRS, and Barthel Index), cognitive and depressive status (MMSE and BDI-II), metabolic syndrome, medication use (antidepressants, anxiolytics, antihypertensives, antidiabetics, and lipid-lowering drugs), alcohol and tobacco use, physical activity levels (VREM), adherence to the Mediterranean diet (MedAS), sleep quality (PSQI), and BMI. All variables corresponded to baseline assessments, but we also considered the change scores for lifestyle variables and BMI. Variables that remained statistically significant after applying FDR correction were incorporated into the final sensitivity ANCOVA models for the corresponding biomarker. This approach allowed us to explore their potential influence on the observed intervention effects.

**Figure 1:**
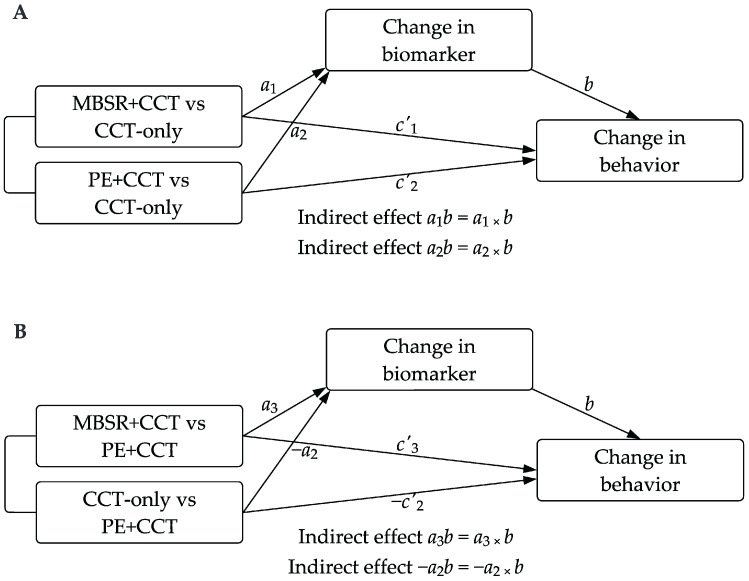
Mediation analysis of intervention effects on behavioral outcomes via biomarker changes. Panel (**A**): MBSR+CCT and PE+CCT are compared to the CCT-only group. Paths *a*_1_ and *a*_2_ represent the intervention effects on the biomarker, while path *b* captures the relationship between the biomarker change and the behavioral outcome. Paths *c*_1_’ and *c*_2_’ illustrate the direct effects of the interventions on the behavioral outcome, controlling for the mediator. Indirect effects are calculated as *a*_1_ × *b* and *a*_2_ × *b*. Panel (**B**): The reference group is changed to PE+CCT, comparing MBSR+CCT and CCT-only against PE+CCT. Path *a*_2_ remains consistent with Panel A but has an inverted sign, resulting in inverted indirect effects (*a*_2_ × *b*). Abbreviations: CCT, computerized cognitive training; MBSR, Mindfulness-Based Stress Reduction; PE, physical exercise.

A secondary objective of our trial was to explore the mechanisms underlying changes in primary outcomes (cognitive and mental health variables) and other behavioral outcomes [70]. We conducted mediation analyses using the PROCESS macro [107] in R, treating groups as multi-categorical dummy-coded variables and alternating the reference group between CCT-only and PE+CCT to perform all pairwise group comparisons (Figure 1). Biomarkers with significant between-group effects in the ANCOVA were identified as potential mediators. Dependent variables included change scores for three cognitive factors, mental health questionnaires (BDI-II, DASS-21), mindfulness measures (MAAS, FFMQ), and physical fitness tests (30CST, 30ACT, 2MST). Following recent recommendations, we tested mediation effects regardless of significant group differences in the dependent variable, as indirect effects may exist even when the total effect is weak or non-significant [108]. We used percentile bootstrap resampling with 10,000 iterations to assess the significance of indirect effects. Since each mediation model involved three pairwise group comparisons, we applied Bonferroni correction to the 95% confidence intervals to account for multiple testing. Mediation was deemed statistically significant if the interval did not include zero. Finally, we examined associations between changes in all biomarkers and behavioral outcomes within each group using pairwise Pearson partial correlations, adjusting for the pre-specified covariates and with FDR correction for multiple testing.

As outlined in the study protocol [70], analyses were conducted using two approaches: the per-protocol approach, which included participants with intervention adherence ≥80%, and the complete-case approach, which approximates the intention-to-treat principle and includes participants with biochemical parameter data available at both time points. Missing data were not imputed, as the overall proportion of missing data was low (8.94% across the sample). Results from the per-protocol sample are presented in the main text, while findings from the complete-case analyses are briefly discussed in the main text and detailed in the Supplementary Materials.

## 3. Results

### 3.1. Participants

**Figure 2:**
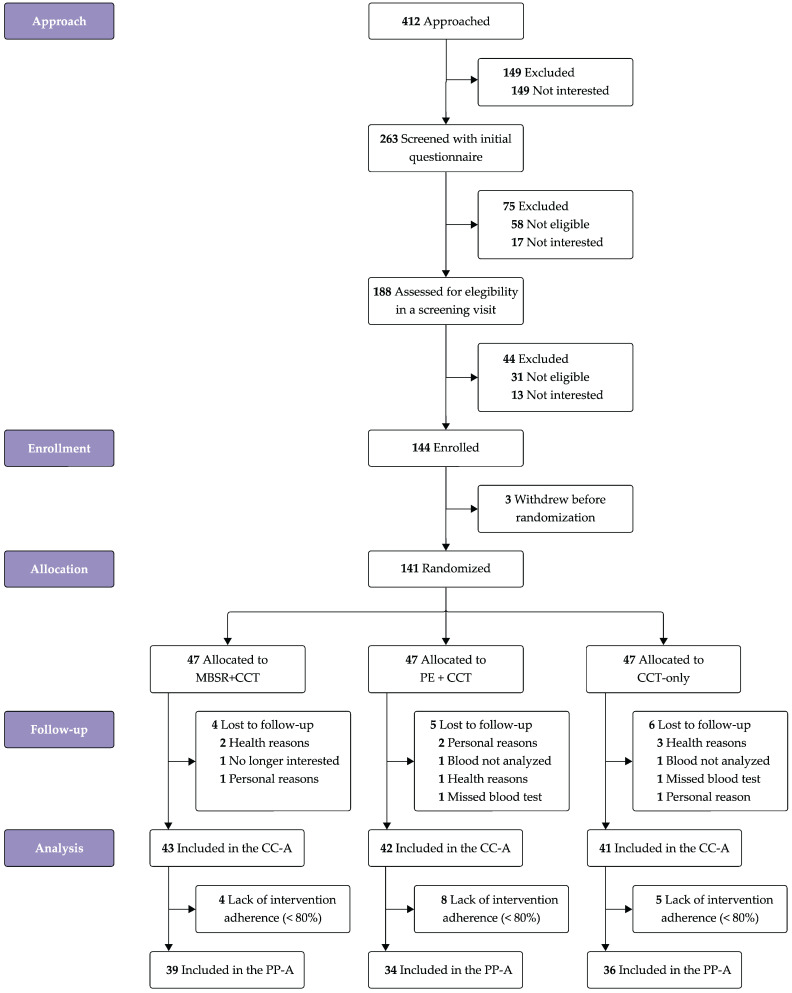
Trial flow diagram. Abbreviations: CC-A, complete-case analysis; CCT, computerized cognitive training; MBSR, Mindfulness-Based Stress Reduction; PE, physical exercise; PP-A, per-protocol analysis.

Out of 412 individuals screened, 141 were randomly assigned to the three intervention arms in a 1:1:1 ratio. Individuals not enrolled were excluded due to disinterest or ineligibility (see Figure 2 for a detailed breakdown of exclusions at each screening stage). Biomarker data loss to follow-up was observed in 15 participants (10.64%): four, five, and six individuals in the MBSR+CCT, PE+CCT, and CCT-only groups, respectively. Reasons for loss to follow-up included health-related issues (unrelated to the interventions), other personal matters, or failure to attend the blood-sample visit. Among the 126 participants with complete biomarker data at both time points, 17 were excluded from the per-protocol analysis due to insufficient adherence to the intervention (<80%).

Table 4 presents the demographic and clinical characteristics of the 109 participants in the per-protocol sample. Overall, the groups were well balanced, with only two variables showing statistically significant between-group differences: time since stroke and depression scores. Specifically, the MBSR+CCT group had a longer post-stroke duration than the CCT-only group, and higher BDI-II depression scores than the PE+CCT group (both *p*_adj_ < 0.05, Bonferroni-corrected). Descriptive statistics for the complete-case sample and the whole sample (i.e., all randomized participants) are provided in Supplementary Materials, Table S3. As shown in Table S4, no significant baseline differences were observed among the per-protocol, the complete-case, and the whole samples. Furthermore, no statistically significant differences were found between participants who remained in the study and those who dropped out (all *p* > 0.05). Based on these analyses, it is reasonable to conclude that these imbalances do not indicate a systematic bias and could plausibly be attributed to chance. Nonetheless, our primary analyses considered these two variables, ensuring their potential influence was adequately controlled (see Section 3.3).

### 3.2. Intervention Adherence and Safety

Adherence rates for each intervention group across the per-protocol and complete-case samples are presented in the Supplementary Materials, Table S5. The mean adherence rates (SD) for the whole sample were as follows: 82.99% (23.20%) for the MBSR+CCT group, 82.13% (21.98%) for the PE+CCT group, and 84.96% (28.36%) for the CCT-only group. Notably, 78.01% of participants (*n* = 110) maintained adherence above the 80% benchmark.

During the intervention period, six adverse events were reported. In the MBSR+CCT group, two events occurred: a toe amputation and hospitalization due to COVID-19. One adverse event was recorded in the PE+CCT group (hemorrhoid surgery). In the CCT-only group, three events were reported: hospitalization for an unspecified illness, ischemic stroke, and death due to a hemorrhagic stroke. No cases of fatigue, pain, or falls were reported. Based on the available data, none of these adverse events were determined to be related to the study interventions.

### 3.3. Between-Group Changes in Biomarkers

ANCOVA models (Table 5) identified a significant between-group difference in VEGF change scores, which remained significant after FDR adjustment. Post hoc Bonferroni-corrected analyses revealed that VEGF concentrations significantly decreased in the PE+CCT and CCT-only groups compared to the MBSR+CCT group. The effect sizes for the ANCOVA and post hoc tests indicated that these differences were of moderate-to-large magnitude according to standard benchmarks [109,110]. No other significant between-group differences were observed for the remaining biomarkers. These findings were consistent with the results of the complete-case analysis (Supplementary Materials, Table S6), although the between-group difference in VEGF changes was only significant before FDR correction (*p* = 0.009, *p*_adj_ = 0.052).

Our primary analyses replaced non-detectable VEGF and CRP values with half the LLOQ. As a sensitivity check, lognormal imputation models were used to estimate biomarker values below the detection limit based on the data distribution. These models verified the consistency of our findings (Supplementary Materials, Table S7).

We conducted Pearson partial correlation analyses to determine whether additional covariates were necessary beyond those pre-specified in the ANCOVA models. Among the variables tested, baseline BMI was the only factor significantly associated with CRP changes after FDR correction in the per-protocol and complete-case samples (Supplementary Materials, Figures S2 and S3). Consequently, BMI was included as a covariate in sensitivity ANCOVA models for CRP. These adjusted models confirmed that intervention effects on CRP were not significant in either the per-protocol sample (*F*_(2,97)_ = 0.685, *p* = 0.506, *η*_p_^2^ = 0.014) or the complete-case sample (*F*_(2,114)_ = 0.760, *p* = 0.470, *η*_p_^2^ = 0.013).

### 3.4. Within-Group Changes in Biomarkers

**Figure 3:**
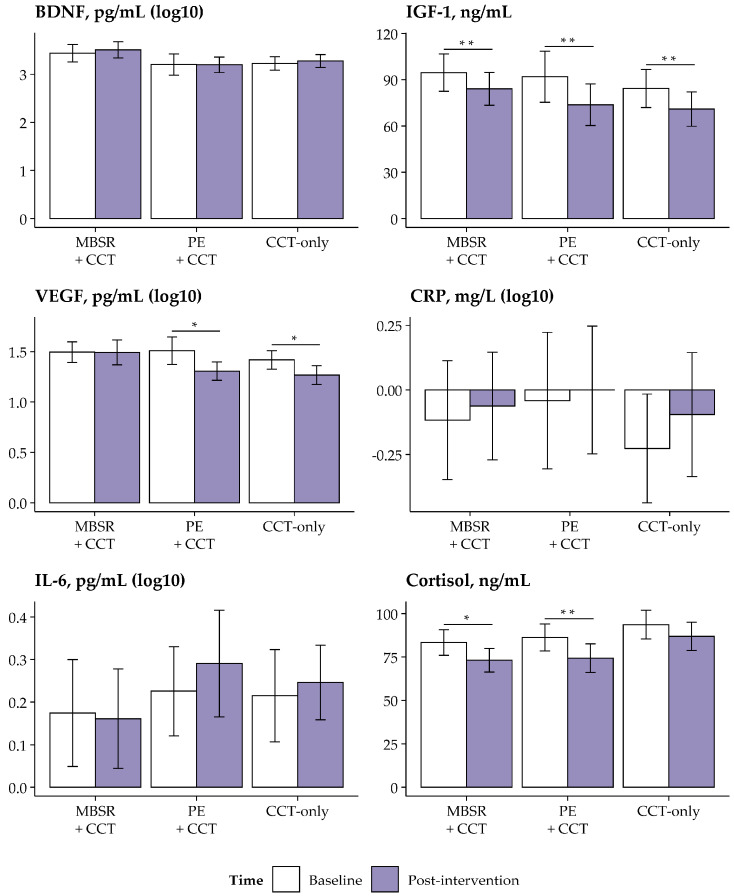
Changes in blood biomarkers across intervention groups (per-protocol sample). Mean levels of biomarkers at baseline (white bars) and post-intervention (purple bars) are shown for the MBSR+CCT, PE+CCT, and CCT-only groups. Error bars represent 95% confidence intervals. Significant within-group changes, adjusted for multiple comparisons using the FDR correction, are marked as follows: * *p* < 0.05, ** *p* < 0.01. Abbreviations: CCT, computerized cognitive training; FDR, false discovery rate; MBSR, Mindfulness-Based Stress Reduction; PE, physical exercise.

Paired *t*-tests analyzed changes in biomarkers within each group (Table 6). The MBSR+CCT group showed a significant reduction in cortisol levels. The PE+CCT group exhibited significant reductions in VEGF and cortisol concentrations. The CCT-only group showed a significant decrease in VEGF levels. IGF-1 levels decreased significantly in all three groups. In contrast, no significant changes were observed for BDNF or inflammatory markers in any group. All significant differences remained after FDR correction and corresponded to medium-to-large effect sizes (*d* = 0.45–0.73). These findings are visually summarized in Figure 3. Results from the complete-case analysis were consistent with the per-protocol analysis (Supplementary Materials, Table S8), and no variations were noted when considering the lognormal distribution imputation method for VEGF and CRP (Supplementary Materials, Table S7).

### 3.5. Mediation and Correlational Analyses

No mediation effects were identified in the per-protocol or complete-case samples regarding the observed between-group differences in VEGF levels on cognitive, mental health, mindfulness, and physical fitness scores (Supplementary Materials, Table S9). Moreover, no significant partial correlations between biomarker changes and behavioral data changes were detected within each group after FDR correction in the per-protocol sample (Supplementary Materials, Figure S4). In the complete-case sample (Supplementary Materials, Figure S5), the only significant correlation was a positive association between increased IGF-1 levels and depression scores measured by the BDI-II in the MBSR+CCT group (*r*_p (34)_ = 0.56, *p*_adj_ = 0.021).

## 4. Discussion

The present study examined between- and within-group changes in growth factors (BDNF, IGF-1, and VEGF), inflammatory markers (CRP, IL-6), and cortisol following the combination of 12-week MBSR or PE programs with CCT in patients with chronic stroke. Additionally, it explored how changes in these biomarkers were associated with cognitive, emotional, mindfulness, and fitness outcomes. Overall, our findings showed the following: (i) a significant between-group difference in VEGF, with levels remaining unchanged in the MBSR+CCT group but significantly decreasing in the PE+CCT and CCT-only groups; (ii) significant within-group reductions in cortisol for MBSR+CCT and PE+CCT, though this did not result in a between-group difference; and (iii) significant decreases in IGF-1 across all groups, with no between-group differences. No significant changes were observed for BDNF, IL-6, or CRP within or between groups. Furthermore, no biomarker changes, except for IGF-1 and depression scores in the MBSR+CCT group, were significantly correlated with or mediated the gains observed in cognitive, emotional, mindfulness, or fitness outcomes. Below, we discuss the implications of these findings, study limitations, and future research directions.

In this study, the most notable result was that participants in the MBSR+CCT group did not exhibit the post-intervention VEGF decline observed in the other two groups. VEGF plays a key role in endothelial function and angiogenesis and has also been linked to neuroplastic processes through its interactions with BDNF and IGF-1 [111]. The cause of the VEGF decline in the PE+CCT and CCT-only groups is unclear. However, because these two groups did not significantly differ from each other, the reduction is unlikely due to the PE component. Instead, it may reflect common factors across all groups, such as individual recovery dynamics (e.g., see [112]), natural fluctuations in VEGF levels, or regression toward the mean. Assuming this general reduction pattern, the MBSR component may have counteracted VEGF declines, although the exact mechanism remains unknown due to the limited prior research on MBIs and VEGF. Only one previous study reported lower VEGF levels in wound fluid after MBSR in healthy men [113], but differences in populations and biological substrates complicate direct comparisons. Therefore, it is useful to consider broader mechanisms that may underlie VEGF regulation, particularly in relation to stress, a primary target of MBIs. Psychological stress impairs endothelial function by raising cortisol and catecholamine levels, enhancing endothelin-1 activity, increasing oxidative stress, and elevating pro-inflammatory cytokines, all of which reduce nitric oxide (NO) availability [114,115,116]. Since NO and VEGF regulate each other [117], stress-induced NO depletion may impair VEGF activity. Indeed, animal studies have provided preliminary evidence of VEGF reductions following exposure to chronic stress [118,119]. In this context, MBSR may have preserved VEGF levels in our study by downregulating stress-related pathways, maintaining endothelial NO synthase activity, sustaining NO bioavailability, and stabilizing VEGF signaling. Although speculative, this hypothesis merits further investigation, given its potential clinical implications for vascular homeostasis, cardiovascular health, and stroke recovery.

In contrast to VEGF, the other two growth factors, BDNF and IGF-1, showed no significant differences between groups. BDNF remained stable across all arms, while IGF-1 decreased significantly in every group, suggesting that this decline was unrelated to MBSR or PE interventions. Our findings contradict a meta-analysis indicating that MBIs may elevate peripheral BDNF in non-stroke populations [52]. However, that analysis included few studies with considerable methodological heterogeneity, making it premature to conclude that mindfulness consistently upregulates BDNF. Similarly, PE+CCT did not increase BDNF, IGF-1, or VEGF. While some trials have reported increases with regular PE programs in chronic stroke [60,61,62], others found no significant effects [25,63,64], and our findings align with the latter. Discrepancies may stem from exercise intensity, as high-intensity aerobic exercise is most effective for increasing BDNF in patients with stroke [65]. Consequently, our moderate-intensity intervention may have been insufficient. The lack of adequate intervention duration may also be a reason (e.g., evidence suggests that circulating IGF-1 increases only in programs lasting more than 12 weeks, although local upregulation may still support early neuromuscular adaptations [120]). The type of exercise is another important consideration. Our intervention combined aerobic and strength training, but the molecular mechanisms underlying resistance exercise are less well understood, and growth factors may be less responsive to it. Resistance training may confer benefits through alternative pathways, such as myokine secretion during muscle contractions, which facilitates communication between muscles and the brain and may support neuroprotection and plasticity [56,121]. Beyond intervention factors, individual characteristics can influence growth factor responses. To account for this, we explored a broad range of baseline factors as potential covariates. However, none significantly predicted growth factor changes after FDR correction, though analyzing these factors individually may have overlooked complex interactions. Unassessed variables, such as stroke etiology and genetic polymorphisms (e.g., *BDNF Val66Met*), may contribute to distinct physiological profiles that influence neuroplasticity and response to exercise [122]. Additionally, methodological differences in biomarker collection should be considered, as plasma and serum measurements can yield different results due to platelet release of BDNF and VEGF during coagulation [123,124].

Regarding stress regulation and inflammation markers, participants in both combined-intervention arms (MBSR+CCT and PE+CCT) experienced significant within-group reductions in cortisol levels, suggesting that MBSR and PE may downregulate stress pathways beyond the effects of CCT alone. This finding, consistent with prior research in other populations [45,47,49,57], is particularly novel in the context of chronic stroke due to the limited research on stress-related biomarker changes following MBIs and PE programs in this population. Clinically, this result may be relevant, as chronically elevated cortisol has been linked to worse post-stroke outcomes [125] and, more broadly, to physiological disruptions that may compromise overall health, including increased inflammation, metabolic dysfunction, heightened cardiovascular risk, and adverse neuropathological changes [126]. However, these cortisol reductions were not large enough to yield statistically significant between-group differences, possibly because the CCT-only group also exhibited modest, though non-significant, cortisol decreases. Such reductions may reflect the general benefits of rehabilitation participation and social engagement with peers, as previously described in a qualitative study by participants in the CCT-only group [72]. In contrast, inflammatory markers (CRP and IL-6) did not appreciably change in any intervention arm. While one PE trial in patients with stroke [66] and mindfulness-based trials in other populations (e.g., [43,44,51]) have reported reductions in inflammatory markers, we did not observe those effects here. As with growth factors, multiple factors, ranging from intervention parameters to baseline participant characteristics, may underlie this absence of change. Nevertheless, the low baseline inflammation in our sample is particularly relevant, as it likely limits the potential for further reductions in CRP or IL-6. The majority of participants (77.78%) had CRP values below the commonly used cutoff of <3 mg/L, while 16.30% had normal to mildly elevated CRP levels (3–10 mg/L) [127]. Similarly, most participants (92.75%) had IL-6 values below the pooled mean of 5.19 pg/mL reported for healthy populations [128].

Despite the observed physiological changes, these shifts neither correlated with nor mediated the behavioral improvements reported in our previous study [71]. A single correlation between decreased IGF-1 and improved depression in the MBSR+CCT group emerged in the complete-case analysis but did not persist in the per-protocol analysis (≥80% adherence). Nonetheless, given IGF-1’s important role in brain function [129] and the high prevalence of post-stroke depression [5,7], further research should explore this relationship. Several factors may explain the absence of reliable biomarker–behavior links, including previously discussed considerations (e.g., insufficient intervention duration, individual moderators) and additional elements. For instance, the behavioral measures used may have been too broad to capture subtle biomarker effects (e.g., VO_2_ max might be more sensitive than general fitness indicators such as those from the SFT). Additionally, although the biomarkers measured were among the most studied in prior research, they may not have been the most responsive to these interventions. Because behavioral interventions likely influence multiple interrelated biological pathways, each involving a cascade of molecular reactions, it is unlikely that any single biomarker fully reflects their complexity. Interpreting peripheral biomarker changes and their correlates is also challenging for at least two reasons. First, peripheral biomarkers provide only an indirect measure of brain-level processes since the blood-brain barrier selectively regulates molecular exchange, leaving it unclear whether peripheral changes accurately mirror central concentrations [59]. Second, biomarkers do not always follow a simple “more is better” or “less is better” framework (e.g., increasing VEGF supports angiogenesis but can also compromise blood–brain barrier integrity [111]). Future research using advanced analytical methods and larger sample sizes could adopt a systems-level approach to analyze multiple biomarker panels and identify subtle clusters that better predict or explain behavioral changes. Neuroimaging could further validate these biological signatures and clarify the role of peripheral changes.

### Strengths and Limitations

This study has several strengths, including its randomized controlled design and integration of multiple biomarkers reflecting distinct physiological processes. Additionally, it is one of the first studies to examine the effects of mindfulness on biomarkers in patients with stroke and compare these effects with those of PE in the same population. We included a wide range of stroke subtypes, which, while introducing some heterogeneity and potentially diluting group-level effects, also reflects the clinical reality of stroke and enhances the study’s ecological validity. Finally, our sample size was relatively large compared to previous stroke trials, which is a notable strength.

Several limitations beyond those previously discussed should be acknowledged. First, we excluded individuals with severe stroke presentations, such as those with pronounced cognitive deficits or aphasia. As a result, our findings may not generalize to these subgroups, which might paradoxically benefit the most from these interventions. Second, the 15-day permitted gap between intervention completion and biomarker sampling may have introduced variability, potentially diluting transient yet important biomarker responses to interventions. Third, the absence of long-term follow-up assessments limits our ability to determine whether biomarker changes emerged later or persisted over time. Fourth, participants in the MBSR+CCT and PE+CCT groups received twice the intervention time of those in the CCT-only group, potentially influencing outcomes. Fifth, the lack of a passive control group further complicates the interpretation of some longitudinal changes and their underlying causes. Finally, since the study was not primarily powered for biomarker outcomes, there remains a risk of type II errors.

## 5. Conclusions

Our findings suggest that incorporating MBSR or PE into a 12-week CCT program for chronic stroke survivors leads to moderate and selective changes in specific peripheral biomarkers. Specifically, mindfulness stabilized VEGF levels, while mindfulness and PE reduced cortisol. In contrast, no significant effects were observed on other growth factors or inflammatory markers. These results suggest that mindfulness and exercise may modulate certain biological processes relevant to stroke recovery, even in the chronic phase—a period typically associated with diminished neuroplasticity and recovery potential. Such biological changes may support brain health and help prevent post-stroke decline or vascular dementia. However, the lack of significant mediation or correlation between biomarker changes and behavioral improvements highlights the need for further research to better understand their clinical implications. Future well-powered studies with longer interventions and follow-up assessments should aim to confirm these findings, investigate individual differences and intervention parameters that may influence treatment effects, and incorporate neuroimaging to elucidate central mechanisms.

## Figures and Tables

**Table 1:** Eligibility criteria.

Inclusion Criteria	Exclusion Criteria
Age between 18–80 years old	Cognitive impairment (MMSE ≤ 23)
Diagnosis of ischemic or hemorrhagic stroke	Severe aphasia (>2 in item 9 in NIHSS)
Stroke diagnosis between 3–60 months ago	Severe sensory problems
Consent from a physician to engage in PE	Diagnosis of transient ischemic attack
Fluency in Spanish or Catalan	Other neurological conditions
	Severe pre-stroke psychiatric disorder
	History of alcohol or substance abuse

Abbreviations: NIHSS, National Institutes of Health Stroke Scale [74]; MMSE, Mini-Mental State Examination [75]; PE, physical exercise.

**Table 2:** Comparison between the standard MBSR schedule and the MindFit program.

Week	MindFit MBSR	Standard MBSR [76]
1	Orientation Session	Orientation Session + Session 1
2	Session 1	Session 2
3	Session 2	Session 3
4	Session 3	Session 4
5	No session	Session 5
6	Session 4	Session 6 + All-day session
7	Session 5	Session 7
8	Session 6	Session 8
9	All-day Session	-
10	No session	-
11	Session 7	-
12	Session 8	-

Note: All weeks involve home practice consisting of formal and informal meditation exercises. Abbreviations: MBSR, Mindfulness-Based Stress Reduction.

**Table 3:** Description of each session type in the PE program.

Type of Session	Sess/wk	Min/sess	Brief Description
Synchronous			
Strength, agility, and balance training	2	60	Sessions included two balance and agility exercises, followed by six strength exercises alternating upper and lower limbs. Sets/reps per exercise: 2 × 10 → 3 × 15 Intensity: Borg RPE scale 12–14/20 (constant across weeks)
Aerobic training	1	45	Low-impact cardiovascular exercises Intensity progression: - Weeks 1–6: Borg RPE scale 11–12/20 - Weeks 7–12: Borg RPE scale 13–14/20
Autonomous			
Walking training	2	45	Walking sessions conducted around participants’ homes Intensity progression: - Weeks 1–2: Borg RPE scale 9–10/20 - Weeks 3–6: Borg RPE scale 11–12/20 - Weeks 7–12: Borg RPE scale 13–14/20

Note: All sessions followed the same structure: warm-up, central part, and cool-down. Intensity levels on the Borg RPE Scale are interpreted as follows: 9–10/20 indicates very light exertion, barely noticeable; 11–12/20 represents light exertion, where activities feel comfortable and sustainable; and 13–14/20 reflects somewhat hard exertion, requiring noticeable effort but remaining manageable throughout the session [78]. According to the American College of Sports Medicine guidelines, an RPE of 9–11/20 corresponds to low intensity, 12–13/20 to moderate intensity, and 14–17/20 to high intensity [79]. Abbreviations: PE, physical exercise; RPE, rate of perceived exertion.

**Table 4:** Baseline characteristics of participants by treatment group (per-protocol sample).

**Variable**	**MBSR+CCT** **(*n* = 39)**	**PE+CCT** **(*n* = 34)**	**CCT-Only** **(*n* = 36)**	***p*** **-Value**
Age, y	55.46 (10.74)	58.80 (11.59)	58.67 (8.57)	0.291
Sex, *n* (%)				0.424
Male	19 (48.70)	20 (58.80)	23 (63.89)	
Female	20 (51.30)	14 (41.20)	13 (36.11)	
Education, y	12.54 (3.02)	13.09 (4.00)	11.58 (3.38)	0.170
Number of strokes	1.05 (0.22)	1.12 (0.48)	1.14 (0.35)	0.356
Time since the stroke, mo	33.96 (21.66)	26.72 (18.91)	21.06 (16.53)	**0.017**
Type of stroke, *n* (%)				0.645
Ischemic stroke	24 (61.50)	26 (76.50)	24 (66.67)	
Intracerebral hemorrhage	11 (28.20)	7 (20.60)	10 (27.78)	
Subarachnoid hemorrhage	4 (10.30)	1 (2.90)	2 (5.56)	
Circulation, *n* (%)				0.219
Anterior	30 (76.92)	21 (61.76)	25 (69.44)	
Posterior	9 (23.08)	13 (38.24)	9 (25.00)	
Unknown	0 (0.00)	0 (0.00)	2 (5.56)	
Brain side affected, *n* (%)				0.119
Right	19 (48.72)	8 (23.53)	17 (47.22)	
Left	15 (38.46)	23 (67.65)	17 (47.22)	
Bilateral	4 (10.26)	3 (8.82)	2 (5.56)	
Unknown	1 (2.56)	0 (0.00)	0 (0.00)	
National Institutes of Health Stroke Scale (0–42)	2.87 (3.33)	2.24 (2.52)	3.11 (3.39)	0.483
Modified Rankin Scale (0–6)	2.26 (1.04)	2.06 (0.89)	2.47 (1.11)	0.297
Barthel Index (0–100)	91.54 (17.48)	95.00 (10.66)	92.50 (14.22)	0.585
Mini-Mental State Examination (0–30)	28.46 (1.71)	28.85 (1.23)	28.56 (1.23)	0.545
WAIS-III - Vocabulary subtest (0–66)	46.69 (11.03)	46.47 (11.28)	43.69 (8.87)	0.398
Beck Depression Inventory-II (0–63)	18.36 (10.09)	11.47 (7.08)	16.53 (9.84)	**0.009**
Body mass index, kg/m^2^	27.16 (4.88)	27.12 (5.63)	27.89 (4.58)	0.768
Metabolic syndrome, *n* (%)				0.369
Presence	18 (46.15)	21 (61.76)	21 (58.33)	
Absence	21 (53.85)	13 (38.24)	15 (41.67)	

Note: Data are presented as M (SD) unless otherwise noted. Statistics are based on the per-protocol sample, including participants with data at both time points and intervention adherence ≥80%. Higher scores on the National Institutes of Health Stroke Scale, the modified Rankin Scale, and the Beck Depression Inventory-II reflect worse outcomes, indicating greater stroke severity, higher levels of disability, and more severe depressive symptoms, respectively. In contrast, higher scores on the Barthel Index, Mini-Mental State Examination, and WAIS-III Vocabulary subtest represent better outcomes, signifying greater independence, better cognitive function, and higher vocabulary proficiency. Between-group comparisons for continuous variables were analyzed using a one-way analysis of variance if the assumptions of homogeneity of variance and normality were met; otherwise, the Kruskal–Wallis test was used. Categorical variables were compared using the chi-square test, except when expected frequencies were <5, in which Fisher’s exact test was applied. Reported *p*-values correspond to the primary statistical test used for each variable. *p*-values < 0.05 are shown in bold. Abbreviations: CCT, computerized cognitive training; MBSR, Mindfulness-Based Stress Reduction; PE, physical exercise; WAIS-III, Wechsler Adult Intelligence Scale-III.3.3.

**Table 5:** Between-group differences in biomarker changes (per-protocol sample).

Biomarker	Group	Difference Score (T_1_ − T_0_)			Omnibus Test				Post Hoc Comparison				
		*n* *	M (SD)	M_adj_ (SE) ^†^	*F*	*p*	*p*_adj_ ^‡^	*η*_p_^2^	Contrast	MD (95% CI) ^§^	*p*	*p*_adj_ ^§^	*d*
Growth factors													
BDNF, pg/mL (log_10_)	MBSR+CCT (G_1_)	39	0.07 (0.42)	0.11 (0.06)	1.69	0.190	0.228	0.03					
	PE+CCT (G_2_)	34	−0.01 (0.45)	−0.04 (0.06)									
	CCT−only (G_3_)	36	0.05 (0.31)	0.04 (0.06)									
IGF-1, ng/mL	MBSR+CCT (G_1_)	39	−10.48 (17.91)	−8.80 (2.96)	2.55	0.083	0.166	0.05					
	PE+CCT (G_2_)	34	−18.21 (24.89)	−18.39 (3.11)									
	CCT−only (G_3_)	36	−13.32 (21.02)	−14.98 (3.09)									
VEGF, pg/mL (log_10_)	MBSR+CCT (G_1_)	39	0.00 (0.45)	0.02 (0.05)	5.31	**0.006**	**0.038**	0.10	G_1_ vs. G_3_	0.22 (0.04, 0.41)	**0.004**	**0.013**	0.71
	PE+CCT (G_2_)	34	−0.20 (0.37)	−0.18 (0.05)					G_2_ vs. G_3_	0.02 (−0.17, 0.21)	0.790	1.000	0.07
	CCT−only (G_3_)	36	−0.15 (0.31)	−0.20 (0.05)					G_1_ vs. G_2_	0.20 (0.02, 0.39)	**0.008**	**0.025**	0.65
Inflammatory markers													
CRP, mg/L (log_10_)	MBSR+CCT (G_1_)	39	0.05 (0.86)	−0.02 (0.10)	0.69	0.506	0.506	0.01					
	PE+CCT (G_2_)	33	0.04 (0.50)	0.11 (0.11)									
	CCT−only (G_3_)	34	0.13 (0.76)	0.15 (0.11)									
IL-6, pg/mL (log_10_)	MBSR+CCT (G_1_)	39	−0.01 (0.37)	−0.04 (0.04)	1.91	0.154	0.228	0.04					
	PE+CCT (G_2_)	34	0.06 (0.21)	0.07 (0.04)									
	CCT−only (G_3_)	36	0.03 (0.23)	0.05 (0.04)									
Stress markers													
Cortisol, ng/mL	MBSR+CCT (G_1_)	39	−10.25 (22.62)	−13.60 (3.37)	2.89	0.060	0.166	0.05					
	PE+CCT (G_2_)	34	−11.88 (19.36)	−12.52 (3.53)									
	CCT−only (G_3_)	36	−6.81 (27.39)	−2.59 (3.52)									

Note: Unadjusted and adjusted *p*-values below 0.050 are in bold. * Statistics are based on the per-protocol sample, including participants with data at both time points and intervention adherence ≥80%. The sample size for CRP is slightly smaller because concentrations could not be quantified in three cases (two PE+CCT, one CCT-only) due to insufficient samples. ^†^ Marginal means are adjusted for sex, age, years of education, time since stroke, and baseline biomarker values. ^‡^ *F*-test significance levels were FDR-corrected. ^§^ The 95% CI for post hoc mean differences and adjusted *p*-values were Bonferroni-corrected. Abbreviations: BDNF, brain-derived neurotrophic factor; CCT, computerized cognitive training; CRP, C-reactive protein; FDR, false discovery rate; IGF-1, insulin-like growth factor-1; IL-6, interleukin-6; MBSR, Mindfulness-Based Stress Reduction; PE, physical exercise; VEGF, vascular endothelial growth factor.

**Table 6:** Within-group changes in biomarker concentrations (per-protocol sample).

Biomarker	Group	*n* *	Baseline (T_0_) M (SD)	Post-int. (T_1_) M (SD)	Difference Score (T_1_ − T_0_)		*t*	*p*	*p*_adj_ ^†^	*d*
					M (SD)	95% CI ^†^				
Growth factors										
BDNF, pg/mL (log_10_)	MBSR+CCT	39	3.44 (0.57)	3.51 (0.54)	0.07 (0.42)	−0.10, 0.23	1.02	0.316	0.493	0.16
	PE+CCT	34	3.20 (0.65)	3.20 (0.48)	−0.01 (0.45)	−0.19, 0.18	−0.09	0.930	0.975	−0.02
	CCT-only	36	3.22 (0.43)	3.28 (0.41)	0.05 (0.31)	−0.08, 0.18	0.99	0.329	0.493	0.17
IGF-1, ng/mL	MBSR+CCT	39	94.58 (38.62)	84.10 (34.07)	−10.48 (17.91)	−17.48, −3.49	−3.66	**<0.001**	**0.005**	−0.59
	PE+CCT	34	91.96 (49.19)	73.75 (40.17)	−18.21 (24.89)	−28.70, −7.73	−4.27	**<0.001**	**0.003**	−0.73
	CCT-only	36	84.32 (37.76)	71.00 (34.21)	−13.32 (21.02)	−21.91, −4.74	−3.80	**<0.001**	**0.005**	−0.63
VEGF, pg/mL (log_10_)	MBSR+CCT	39	1.49 (0.33)	1.49 (0.39)	0.00 (0.45)	−0.18, 0.18	−0.03	0.975	0.975	−0.01
	PE+CCT	34	1.51 (0.40)	1.31 (0.27)	−0.20 (0.37)	−0.36, −0.05	−3.17	**0.003**	**0.012**	−0.54
	CCT-only	36	1.42 (0.28)	1.27 (0.29)	−0.15 (0.31)	−0.28, −0.02	−2.91	**0.006**	**0.019**	−0.49
Inflammatory markers										
CRP, mg/L (log_10_)	MBSR+CCT	39	−0.12 (0.73)	−0.06 (0.66)	0.05 (0.86)	−0.28, 0.39	0.40	0.692	0.831	0.06
	PE+CCT	33	−0.04 (0.78)	0.00 (0.72)	0.04 (0.50)	−0.17, 0.26	0.47	0.639	0.821	0.08
	CCT-only	34	−0.23 (0.63)	−0.10 (0.71)	0.13 (0.76)	−0.19, 0.45	1.01	0.320	0.493	0.17
IL-6, pg/mL (log_10_)	MBSR+CCT	39	0.17 (0.40)	0.16 (0.37)	−0.01 (0.37)	−0.16, 0.13	−0.22	0.824	0.926	−0.04
	PE+CCT	34	0.23 (0.31)	0.29 (0.37)	0.06 (0.21)	−0.02, 0.15	1.84	0.074	0.167	0.32
	CCT-only	36	0.21 (0.33)	0.25 (0.27)	0.03 (0.23)	−0.06, 0.12	0.82	0.416	0.576	0.14
Stress markers										
Cortisol, ng/mL	MBSR+CCT	39	83.36 (23.50)	73.11 (21.62)	−10.25 (22.62)	−19.09, −1.41	−2.83	**0.007**	**0.019**	−0.45
	PE+CCT	34	86.22 (23.05)	74.33 (24.65)	−11.88 (19.36)	−20.04, −3.73	−3.58	**0.001**	**0.005**	−0.61
	CCT-only	36	93.67 (25.28)	86.85 (24.94)	−6.81 (27.39)	−17.99, 4.37	−1.49	0.145	0.289	−0.25

Note: Unadjusted and adjusted *p*-values below 0.05 are in bold. * Statistics are based on the per-protocol sample, including participants with data at both time points and intervention adherence ≥80%. The sample size for CRP is slightly smaller because concentrations could not be quantified in three cases (two PE+CCT, one CCT-only) due to insufficient samples. ^†^ The 95% CI and *p*-values were FDR-corrected. Abbreviations: BDNF, brain-derived neurotrophic factor; CCT, computerized cognitive training; CRP, C-reactive protein; FDR, false discovery rate; IGF-1, insulin-like growth factor-1; IL-6, interleukin-6; MBSR, Mindfulness-Based Stress Reduction; PE, physical exercise; Post-int., post-intervention; VEGF, vascular endothelial growth factor.

## Data Availability

The data presented in this study are available on request from the corresponding author. The data are not publicly available due to privacy restrictions.

## Supplementary Materials

The following supporting information can be downloaded at: https://www.mdpi.com/article/10.3390/jcm14082580/s1, Table S1: CONSORT 2010 checklist; Table S2: Exploratory factor analysis of cognitive assessment scores at baseline (*n* = 141); Table S3: Baseline characteristics of participants by treatment group (complete-case and whole samples); Table S4: Statistical comparisons across per-protocol, complete-case, and whole samples; Table S5: Intervention adherence across groups and samples; Table S6: Between-group differences in biomarker changes (complete-case sample); Table S7: Sensitivity analyses of VEGF and CRP changes with lognormal imputation (per-protocol and complete-case samples); Table S8: Within-group changes in biomarker concentrations (complete-case sample); Table S9: Mediation analyses of VEGF changes in relation to cognitive, mental health, mindfulness, and fitness outcomes (per-protocol and complete-case samples); Figure S1: Biomarker concentration distribution before and after log_10_ transformation; Figure S2: Partial correlation matrices of biomarker changes with clinical, medication, and lifestyle factors (per-protocol sample); Figure S3: Partial correlation matrices of biomarker changes with clinical, medication, and lifestyle factors (complete-case sample); Figure S4: Partial correlation matrices of biomarker changes with behavioral outcomes stratified by intervention group (per-protocol sample); Figure S5: Partial correlation matrices of biomarker changes with behavioral outcomes stratified by intervention group (complete-case sample).

## Abbreviations

The following abbreviations are used in this manuscript:

ANCOVA	Analysis of Covariance
BDI-II	Beck Depression Inventory-II
BDNF	Brain-Derived Neurotrophic Factor
BMI	Body Mass Index
CCT	Computerized Cognitive Training
CRP	C-Reactive Protein
FDR	False Discovery Rate
HPA	Hypothalamic–Pituitary–Adrenal
IGF-1	Insulin-Like Growth Factor-1
IL-6	Interleukin-6
LLOQ	Lower Limit of Quantification
MBIs	Mindfulness-Based Interventions
MBSR	Mindfulness-Based Stress Reduction
MedAS	Mediterranean Diet Adherence Screener
MMSE	Mini-Mental State Examination
mRS	Modified Rankin Scale
NIHSS	National Institutes of Health Stroke Scale
NGF	Nerve Growth Factor
NO	Nitric Oxide
PE	Physical Exercise
Post-int.	Post-Intervention
PSQI	Pittsburg Sleep Quality Index
RPE	Rate of Perceived Exertion
SDF	Stromal Cell-Derived Factor 1-Alpha (SDF1-α)
SFT	Senior Fitness Test
VEGF	Vascular Endothelial Growth Factor
VREM	Short Version of the Minnesota Leisure Time Physical Activity Questionnaire
WAIS-III	Weschler Adult Intelligence Scale-III
