# Supplementary material for: Effects of Mindfulness and Exercise on Growth Factors, Inflammation, and Stress Markers in Chronic Stroke: The MindFit Project Randomized Clinical Trial
Source: J Clin Med. 2025 Apr 9;14(8):2580. doi: 10.3390/jcm14082580 (PMC12028070; doi:10.3390/jcm14082580)
Supplement: Supplementary file 1 [file jcm-14-02580-s001.zip › jcm-3522367-supplementary.pdf]

Table S1. CONSORT 2010 checklist

Section/Topic	Item No	Item Description	Reported on
**Title and abstract**			
	1a	Identification as a randomised trial in the title	Front matter, Title
	1b	Structured summary of trial design, methods, results, and conclusions	Front matter, Abstract
**Introduction**			
Background and objectives	2a	Scientific background and explanation of rationale	Section 1
	2b	Specific objectives or hypotheses	Section 1
**Methods**			
Trial design	3a	Description of trial design (such as parallel, factorial) including allocation ratio	Section 2.1
	3b	Important changes to methods after trial commencement (such as eligibility criteria), with reasons	Section 2.3
Participants	4a	Eligibility criteria for participants	Section 2.3
	4b	Settings and locations where the data were collected	Sections 2.1, 2.7
Interventions	5	The interventions for each group with sufficient details to allow replication, including how and when they were actually administered	Section 2.5
Outcomes	6a	Completely defined pre-specified primary and secondary outcome measures, including how and when they were assessed	Section 2.7
	6b	Any changes to trial outcomes after the trial commenced, with reasons	Section 2.7.2
Sample size	7a	How sample size was determined	Section 2.8
	7b	When applicable, explanation of any interim analyses and stopping guidelines	Not applicable
Randomisation:			
Sequence generation	8a	Method used to generate the random allocation sequence	Section 2.4
	8b	Type of randomisation; details of any restriction (such as blocking and block size)	Section 2.4
Allocation concealment mechanism	9	Mechanism used to implement the random allocation sequence (such as sequentially numbered containers), describing any steps taken to conceal the sequence until interventions were assigned	Section 2.4
Implementation	10	Who generated the random allocation sequence, who enrolled participants, and who assigned participants to interventions	Section 2.4
Blinding	11a	If done, who was blinded after assignment to interventions (for example, participants, care providers, those assessing outcomes) and how	Section 2.4
	11b	If relevant, description of the similarity of interventions	Not applicable

(Continued on the next page)

Table S1. (Continued)

Section/Topic	Item No	Item Description	Reported on
**Methods**			
Statistical methods	12a	Statistical methods used to compare groups for primary and secondary outcomes	Section 2.8
	12b	Methods for additional analyses, such as subgroup analyses and adjusted analyses	Section 2.8; Figure 1
**Results**			
Participant flow	13a	For each group, the numbers of participants who were randomly assigned, received intended treatment, and were analysed for the primary outcome	Section 3.1; Figure 2
	13b	For each group, losses and exclusions after randomisation, together with reasons	Section 3.1; Figure 2
Recruitment	14a	Dates defining the periods of recruitment and follow-up	Sections 2.1 and 2.2
	14b	Why the trial ended or was stopped	Not applicable
Baseline data	15	A table showing baseline demographic and clinical characteristics for each group	Tables 4, S3
Numbers analysed	16	For each group, number of participants (denominator) included in each analysis and whether the analysis was by original assigned groups	Tables 4, 5, S6, S7, S8
Outcomes and estimation	17a	For each primary and secondary outcome, results for each group, and the estimated effect size and its precision (such as 95% confidence interval)	Sections 3.3, 3.4; Tables 4, 5, S6, S8; Figure 3
	17b	For binary outcomes, presentation of both absolute and relative effect sizes is recommended	Not applicable
Ancillary analyses	18	Results of any other analyses performed, including subgroup analyses and adjusted analyses, distinguishing pre-specified from exploratory	Section 3.3, 3.4, 3.5; Tables S8, S9; Figures S2, S3, S4, S5
Harms	19	All important harms or unintended effects in each group	Section 3.2
**Discussion**			
Limitations	20	Trial limitations, addressing sources of potential bias, imprecision, and, if relevant, multiplicity of analyses	Section 4.1
Generalisability	21	Generalisability (external validity, applicability) of the trial findings	Sections 4 and 5
Interpretation	22	Interpretation consistent with results, balancing benefits and harms, and considering other relevant evidence	Sections 4 and 5
**Other information**			
Registration	23	Registration number and name of trial registry	Section 2.1
Protocol	24	Where the full trial protocol can be accessed, if available	Section 2.1
Funding	25	Sources of funding and other support (such as supply of drugs), role of funders	Back matter, Funding

Table S2. Exploratory factor analysis of cognitive assessment scores at baseline (n = 141)

Variable	3-factor solution			Communalities
	**Factor 1**	**Factor 2**	**Factor 3**	
Stroop – Color	**0.91**	0.00	−0.02	0.80
Stroop – Word	**0.83**	0.03	−0.05	0.66
Stroop – Color-Word	**0.71**	0.06	0.16	0.67
Verbal Fluency (PMR)	**0.69**	0.17	0.01	0.57
Verbal Fluency (animals)	**0.69**	0.20	0.04	0.61
WAIS-III – Digits Forward	**0.58**	−0.10	−0.02	0.31
WAIS-III – Digits Backward	**0.50**	−0.13	0.27	0.43
RAVLT – Delayed	−0.07	**0.96**	0.02	0.90
RAVLT – Immediate	−0.04	**0.92**	0.09	0.88
RAVLT – Learning	0.23	**0.78**	−0.02	0.73
ROCF – Copy Accuracy	−0.02	−0.01	**0.78**	0.59
Trail Making Test – Part A	0.11	−0.07	**0.78**	0.68
WAIS-III – Matrix Reasoning	−0.10	−0.01	**0.76**	0.50
ROCF – Memory Accuracy	−0.07	0.12	**0.67**	0.47
Trail Making Test – Part B	0.23	0.00	**0.64**	0.62
WAIS-III – Symbol Coding	0.23	0.10	**0.61**	0.61
Initial eigenvalue	6.92	2.22	1.86	
% explained variance	43.23	13.87	11.62	
Rotation sum of squares loading	5.30	3.23	4.89	

Note: Exploratory factor analysis was conducted on baseline cognitive assessment scores (n = 141) using principal axis factoring with direct oblimin rotation. Parallel analysis supported a three-factor solution, explaining 68.72% of the variance. These factors were labeled Verbal Executive Functioning, Verbal Memory, and Visual Executive Functioning. Variables with factor loadings ≥0.40 were considered salient and are presented in bold. The Boston Naming Test-15 was initially considered but was excluded from the final analysis due to low communality. The model demonstrated excellent sampling adequacy (Kaiser-Meyer-Olkin's test = 0.89; Bartlett's test: $\chi^2_{(136)}$ = 1441.3, p < 0.001). Factor scores were computed using the regression method, and follow-up scores were calculated using baseline coefficients. A sensitivity analysis confirmed that the factor structure remained stable in the follow-up data (n = 107). Abbreviations: ROCF, Rey-Osterrieth Complex Figure; WAIS-III, Wechsler Adult Intelligence Scale-III.

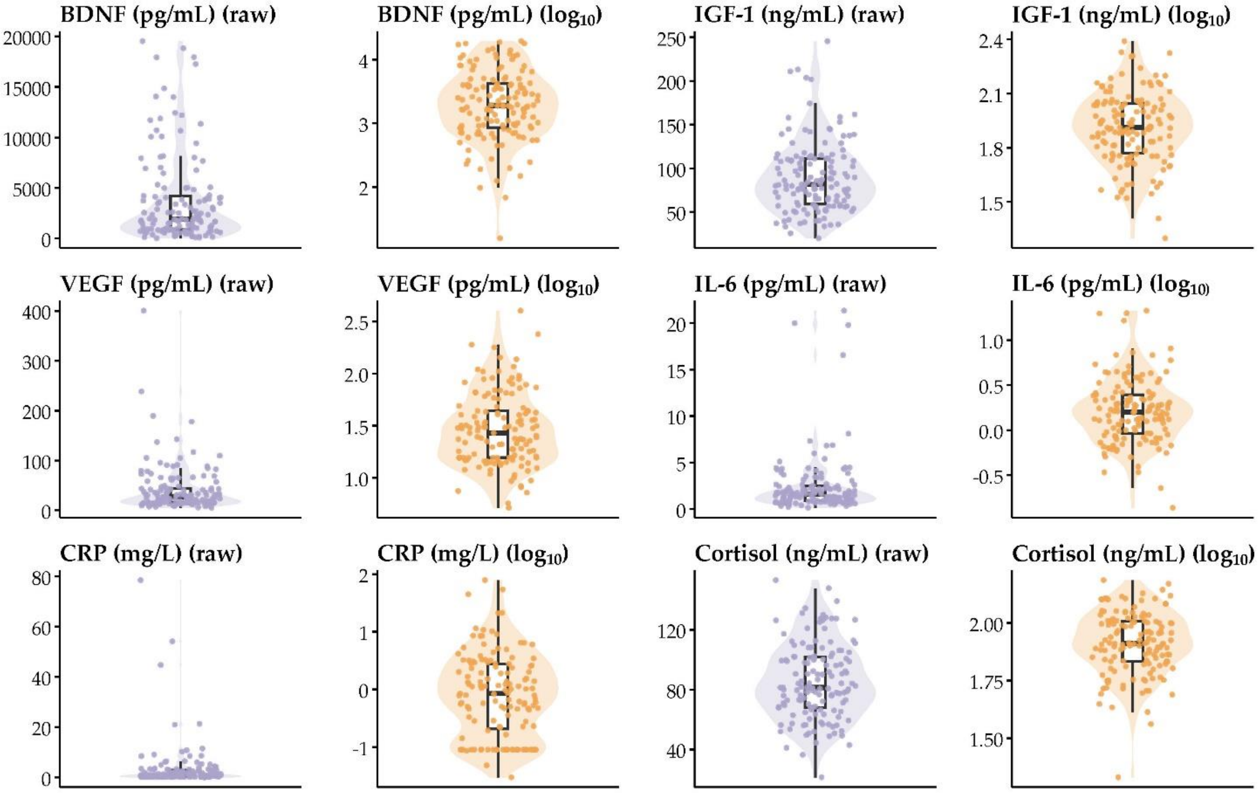

Figure S1. Biomarker concentration distribution before and after log_{10} transformation. Violin plots show raw (left) and log-transformed (right) values for BDNF, IGF-I, VEGF, CRP, IL-6, and cortisol. Embedded box plots indicate the median and interquartile range, illustrating reduced skewness and improved normalization after transformation for BDNF, VEGF, CRP, and IL-6. Abbreviations: BDNF, brain-derived neurotrophic factor; CRP, C-reactive protein; IGF-I, insulin-like growth factor I; IL-6, interleukin-6; VEGF, vascular endothelial growth factor.

Table S3. Baseline characteristics of participants by treatment group (complete-case and whole samples)

Variable		Complete-case sample (*n* = 126)			Whole sample (*n* = 141)		
		**MBSR+CCT (*n* = 43)**	**PE+CCT (*n* = 42)**	**CCT-only (*n* = 41)**	**MBSR+CCT (*n* = 47)**	**PE+CCT (*n* = 47)**	**CCT-only (*n* = 47)**
Age, y		55.67 (11.94)	59.30 (11.52)	57.56 (10.10)	55.93 (11.60)	58.87 (12.03)	58.43 (9.95)
Sex, n (%)	Male	22 (51.16)	26 (61.90)	27 (65.85)	25 (53.19)	29 (61.70)	31 (65.96)
	Female	21 (48.84)	16 (38.10)	14 (34.15)	22 (46.81)	18 (38.30)	16 (34.04)
Education, y		12.72 (3.16)	12.79 (3.94)	11.73 (3.23)	12.45 (3.16)	12.79 (3.86)	11.79 (3.39)
Number of strokes		1.05 (0.21)	1.19 (0.55)	1.12 (0.33)	1.04 (0.20)	1.19 (0.54)	1.15 (0.36)
Time since the stroke, mo		33.36 (22.05)	28.43 (19.61)	22.50 (17.15)	33.36 (21.84)	30.06 (20.32)	22.21 (17.43)
Type of stroke, n (%)	Ischemic	28 (65.12)	30 (71.43)	27 (65.85)	32 (68.09)	33 (70.21)	33 (70.21)
	ICH	11 (25.58)	9 (21.43)	12 (29.27)	11 (23.40)	10 (21.28)	12 (25.53)
	SAH	4 (9.30)	3 (7.14)	2 (4.88)	4 (8.51)	4 (8.51)	2 (4.26)
Circulation, n (%)	Anterior	33 (76.74)	27 (64.29)	27 (65.85)	36 (76.60)	31 (65.96)	32 (68.09)
	Posterior	10 (23.26)	15 (35.71)	11 (26.83)	11 (23.40)	16 (34.04)	12 (25.53)
	Unknown	0 (0.00)	0 (0.00)	3 (7.32)	0 (0.00)	0 (0.00)	3 (6.38)
Brain side affected, n (%)	Right	21 (48.84)	12 (28.57)	20 (48.78)	24 (51.06)	15 (31.91)	24 (51.07)
	Left	16 (37.21)	26 (61.91)	18 (43.90)	17 (36.17)	26 (55.32)	20 (42.55)
	Bilateral	5 (11.63)	4 (9.52)	3 (7.32)	5 (10,64)	5 (10,64)	3 (6.38)
	Unknown	1 (2.32)	0 (0.00)	0 (0.00)	1 (2.13)	1 (2.13)	0 (0.00)
National Institute of Health Stroke Scale (0–42)		2.79 (3.26)	2.26 (2.49)	2.90 (3.26)	2.57 (3.20)	2.32 (2.53)	3.06 (3.41)
Modified Rankin Scale (0–6)		2.30 (1.10)	2.05 (0.91)	2.39 (1.09)	2.26 (1.09)	2.06 (0.89)	2.36 (1.13)
Barthel Index (0–100)		90.47 (18.80)	94.88 (10.50)	93.41 (13.53)	90.85 (18.07)	95.11 (10.13)	92.98 (13.94)
Mini-Mental State Examination (0–30)		28.42 (1.75)	28.71 (1.35)	28.63 (1.18)	28.40 (1.73)	28.72 (1.30)	28.57 (1.33)
WAIS-III – Vocabulary subtest (0–66)		46.74 (10.67)	46.31 (10.21)	44.49 (8.70)	46.64 (10.29)	46.94 (10.03)	44.36 (8.88)
Beck Depression Inventory-II (0–63)		18.61 (10.49)	11.12 (7.30)	16.73 (10.11)	18.33 (10.33)	12.34 (8.52)	15.98 (9.79)
Body mass index, kg/m^2		26.86 (4.88)	26.95 (5.26)	28.06 (4.80)	26.99 (4.84)	27.14 (5.32)	28.11 (4.91)
Metabolic syndrome, n (%)	Presence	21 (48.84)	23 (54.76)	25 (60.98)	23 (48.94)	26 (55.32)	29 (61.70)
	Absence	22 (51.16)	19 (45.24)	16 (39.02)	24 (51.06)	21 (44.68)	18 (38.30)

Note: Data are presented as M (SD) unless otherwise noted. Abbreviations: CCT, computerized cognitive training; ICH, intracerebral hemorrhage; MBSR, Mindfulness-Based Stress Reduction; PE, physical exercise; SHA, subarachnoid hemorrhage; WAIS-III, Wechsler Adult Intelligence Scale-III.

Table S4. Statistical comparisons across per-protocol, complete-case, and whole samples

Variable		Per-protocol (n = 109)	Complete-case (n = 126)	Whole (n = 141)	Statistic	p-Value
Age, y		57.74 (11.22)	57.69 (11.26)	57.56 (10.39)	0.01	0.992
Sex, n (%)	Male	62 (56.88)	75 (59.52)	85 (60.28)	0.31	0.857
	Female	47 (43.12)	51 (40.48)	56 (39.72)		
Education, y		12.34 (3.48)	12.44 (3.51)	12.39 (3.49)	0.03	0.971
Number of strokes		1.13 (0.39)	1.13 (0.41)	1.10 (0.36)	0.24	0.783
Time since the stroke, mo		28.54 (20.35)	27.72 (20.05)	27.44 (19.80)	0.10	0.901
Type of stroke, n (%)	Ischemic	74 (67.89)	86 (68.26)	98 (69.50)	0.21	0.994
	ICH	28 (25.69)	31 (24.60)	33 (23.41)		
	SAH	7 (6.42)	9 (7.14)	10 (7.09)		
Circulation, n (%)	Anterior	76 (69.72)	87 (69.05)	99 (70.21)	0.27	0.999
	Posterior	31 (28.44)	36 (28.57)	39 (27.66)		
	Unknown	2 (1.84)	3 (2.38)	3 (2.13)		
Brain side affected, n (%)	Right	44 (40.37)	53 (42.06)	63 (44.68)	1.34	0.983
	Left	55 (50.46)	60 (47.62)	63 (44.68)		
	Bilateral	9 (8.25)	12 (9.53)	13 (9.22)		
	Unknown	1 (0.92)	1 (0.79)	2 (1.42)		
National Institute of Health Stroke Scale (0–42)		2.65 (3.06)	2.63 (3.02)	2.75 (3.11)	0.05	0.948
Modified Rankin Scale (0–6)		2.23 (1.04)	2.23 (1.05)	2.27 (1.02)	0.05	0.951
Barthel Index (0–100)		92.98 (14.42)	92.90 (14.73)	92.94 (14.49)	0.00	0.999
Mini-Mental State Examination (0–30)		28.57 (1.46)	28.59 (1.45)	28.61 (1.42)	0.03	0.968
WAIS-III – Vocabulary subtest (0–66)		45.98 (9.75)	45.95 (9.91)	45.63 (10.44)	0.04	0.958
Beck Depression Inventory-II (0-63)		15.46 (9.80)	15.19 (9.66)	15.61 (9.54)	0.06	0.945
Body mass index, kg/m^2		27.41 (5.02)	27.28 (4.94)	27.39 (5.00)	0.03	0.973
Metabolic syndrome, n (%)	Presence	60 (55.05)	69 (54.76)	78 (55.32)	0.01	0.995
	Absence	49 (44.95)	57 (45.24)	63 (44.68)		

Note: Data are presented as M (SD) unless otherwise noted. For quantitative variables, the statistical test used is an ANOVA *F*-test. For categorical variables, a Pearson χ^2 test was applied or a Fisher's exact test when expected frequencies were less than 5. Abbreviations: CCT, computerized cognitive training; ICH, intracerebral hemorrhage; MBSR, Mindfulness-Based Stress Reduction; PE, physical exercise; SHA, subarachnoid hemorrhage; WAIS-III, Wechsler Adult Intelligence Scale-III.

Table S5. Intervention adherence across groups and samples

Intervention	Per-protocol sample (*n* = 109)			Complete-case sample (*n* = 126)			Whole sample (*n* = 141)		
	MBSR+CCT (*n* = 39)	PE+CCT (*n* = 34)	CCT-only (*n* = 36)	MBSR+CCT (*n* = 43)	PE+CCT (*n* = 42)	CCT-only (*n* = 41)	MBSR+CCT (*n* = 47)	PE+CCT (*n* = 47)	CCT-only (*n* = 47)
**MBSR**									
Orientation session, n attendees (%)	39 (100.00)			40 (93.02)			41 (87.23)		
No. of guided sessions (0–8)	7.59 (0.82)			7.19 (1.80)			6.81 (2.34)		
All-day session, n attendees (%)	39 (100.00)			40 (93.02)			40 (85.11)		
No. of home practice sessions (0–48)	33.49 (12.26)			31.09 (14.50)			29.21 (15.87)		
No. of hours (0–45)	39.14 (4.66)			36.70 (9.77)			34.45 (12.56)		
Adherence (0–100)	86.97 (10.36)			81.56 (21.71)			76.56 (27.90)		
**PE**									
No. of sessions (0–60)		55.85 (4.35)			52.60 (11.24)			49.17 (15.10)	
Strength sessions (0–24)		21.82 (3.35)			20.74 (4.91)			19.17 (6.74)	
Aerobic sessions (0–12)		11.38 (0.99)			10.67 (2.52)			10.02 (3.15)	
Autonomous walking sessions (0–24)		22.65 (2.07)			21.19 (5.13)			19.98 (6.26)	

(Continued on the next page)

Table S5. (Continued)

Intervention	Per-protocol sample (*n* = 109)			Complete-case sample (*n* = 126)			Whole sample (*n* = 141)		
	MBSR+CCT (*n* = 39)	PE+CCT (*n* = 34)	CCT-only (*n* = 36)	MBSR+CCT (*n* = 43)	PE+CCT (*n* = 42)	CCT-only (*n* = 41)	MBSR+CCT (*n* = 47)	PE+CCT (*n* = 47)	CCT-only (*n* = 47)
**PE**									
Adherence (0–100)		93.09 (7.25)			87.66 (18.73)			81.95 (25.17)	
**CCT**									
No. of tasks	775.90 (159.68)	738.76 (153.45)	791.39 (141.33)	758.77 (174.70)	671.74 (239.19)	714.54 (253.11)	719.47 (230.58)	634.89 (255.77)	682.77 (287.29)
No. of sessions (0–60)	57.92 (4.06)	56.74 (4.94)	58.36 (3.66)	56.74 (6.20)	51.71 (14.33)	53.98 (13.68)	53.66 (13.39)	49.38 (15.94)	50.98 (17.02)
Adherence (0–100)	96.54 (6.77)	94.56 (8.24)	97.27 (6.10)	94.57 (10.34)	86.19 (23.88)	89.96 (22.80)	89.43 (22.32)	82.30 (26.56)	84.96 (28.36)
**Global**									
Adherence (0–100)	91.75 (6.57)	93.82 (5.62)	97.27 (6.10)	88.07 (13.45)	86.92 (16.55)	89.96 (22.80)	82.99 (23.20)	82.13 (21.98)	84.96 (28.36)
Adherence, n ≥ 80% (%)	39 (100.00)	34 (100.00)	36 (100.00)	39 (90.70)	34 (80.95)	36 (87.80)	39 (82.98)	34 (72.34)	37 (78.72)

Note: Data presented as M (SD) unless otherwise indicated. Abbreviations: CCT, computerized cognitive training; MBSR, Mindfulness-Based Stress Reduction; PE, physical exercise.

Table S6. Between-group differences in biomarker changes (complete-case sample)

Biomarker	Group	Difference score ($T_1 - T_0$)			Omnibus test				Post-hoc comparison				
		n *	M (SD)	M_{adj} (SE) †	*F*	*p*	p_{adj} ‡	η_p^2	Contrast	MD (95% CI) §	*p*	p_{adj} §	*d*
**Growth factors**													
BDNF, pg/mL (log_{10})	MBSR+CCT (G_1)	43	0.07 (0.40)	0.10 (0.05)	2.78	0.066	0.182	0.05					
	PE+CCT (G_2)	42	−0.05 (0.42)	−0.07 (0.05)									
	CCT−only (G_3)	41	0.04 (0.31)	0.04 (0.05)									
IGF-1, ng/mL	MBSR+CCT (G_1)	43	−14.03 (23.24)	−11.66 (3.11)	0.52	0.596	0.596	0.01					
	PE+CCT (G_2)	42	−15.03 (26.01)	−15.63 (3.08)									
	CCT−only (G_3)	41	−13.72 (20.10)	−15.60 (3.21)									
VEGF, pg/mL (log_{10})	MBSR+CCT (G_1)	43	0.02 (0.47)	0.03 (0.05)	4.95	**0.009**	0.052	0.08	G_1 vs G_3	0.22 (0.04, 0.40)	**0.003**	**0.009**	0.70
	PE+CCT (G_2)	42	−0.14 (0.41)	−0.12 (0.05)					G_2 vs G_3	0.06 (−0.11, 0.24)	0.363	1.000	0.21
	CCT−only (G_3)	41	−0.16 (0.31)	−0.19 (0.05)					G_1 vs G_2	0.16 (−0.01, 0.33)	**0.028**	0.084	0.49
**Inflammatory markers**													
CRP, mg/L (log_{10})	MBSR+CCT (G_1)	43	0.02 (0.91)	−0.05 (0.09)	0.91	0.406	0.487	0.02					
	PE+CCT (G_2)	41	0.03 (0.53)	0.07 (0.09)									
	CCT−only (G_3)	39	0.09 (0.76)	0.13 (0.10)									
IL-6, pg/mL (log_{10})	MBSR+CCT (G_1)	43	−0.02 (0.36)	−0.05 (0.04)	2.22	0.113	0.182	0.04					
	PE+CCT (G_2)	42	0.03 (0.22)	0.04 (0.04)									
	CCT−only (G_3)	41	0.04 (0.22)	0.07 (0.04)									
**Stress markers**													
Cortisol, ng/mL	MBSR+CCT (G_1)	43	−10.77 (25.22)	−13.16 (3.22)	2.15	0.121	0.182	0.04					
	PE+CCT (G_2)	42	−10.71 (19.01)	−11.36 (3.20)									
	CCT−only (G_3)	41	−7.07 (25.93)	−3.89 (3.32)									

Note: Unadjusted and adjusted *p*-values below 0.05 are in bold. * Statistics are based on the complete-case sample, including participants with data at both time points, regardless of intervention adherence. The sample size for CRP is slightly smaller because concentrations could not be quantified in three cases (two PE+CCT, one CCT-only) due to insufficient samples. † Marginal means are adjusted for sex, age, years of education, time since stroke, and baseline biomarker values. ‡ *F*-test significance levels were FDR-corrected. § The 95% CI for post-hoc mean differences and their *p*-values were Bonferroni-corrected. Abbreviations: BDNF, brain-derived neurotrophic factor; CCT, computerized cognitive training; CRP, C-reactive protein; FDR, false discovery rate; IGF-1, insulin-like growth factor-1; IL-6, interleukin-6; MBSR, Mindfulness-Based Stress Reduction; PE, physical exercise; VEGF, vascular endothelial growth factor.

Table S7. Sensitivity analyses of VEGF and CRP changes with lognormal imputation (per-protocol and complete-case samples)

Biomarker	Sample	n	Baseline (T_0)	Post-int (T_1)	t	p	Difference score ($T_1 - T_0$)		Omnibus test		Post-hoc comparison		
			M (SE)	M (SE)			M (SE)	M_{adj} (SE)	F	p	Contrast	MD (95% CI)	p_{adj}
VEGF (log_{10})	Per-protocol												
	MBSR+CCT (G_1)	39	1.49 (0.05)	1.49 (0.06)	-0.03	0.975	0.00 (0.07)	0.02 (0.05)	4.93	**0.007**	G_1 vs G_3	0.22 (0.03, 0.41)	**0.014**
	PE+CCT (G_2)	34	1.50 (0.07)	1.31 (0.05)	-2.85	0.004	-0.19 (0.07)	-0.17 (0.05)			G_2 vs G_3	0.02 (-0.16, 0.21)	1.000
	CCT−only (G_3)	36	1.42 (0.05)	1.27 (0.05)	-2.83	0.005	-0.15 (0.05)	-0.20 (0.06)			G_1 vs G_2	0.20 (0.01, 0.38)	**0.029**
	Complete-case												
	MBSR+CCT (G_1)	43	1.46 (0.05)	1.48 (0.06)	0.31	0.757	0.02 (0.07)	0.03 (0.05)	4.65	**0.010**	G_1 vs G_3	0.22 (0.04, 0.40)	**0.009**
	PE+CCT (G_2)	42	1.47 (0.06)	1.34 (0.05)	−2.09	0.036	−0.14 (0.07)	−0.12 (0.05)			G_2 vs G_3	0.06 (−0.11, 0.24)	1.000
	CCT−only (G_3)	41	1.43 (0.05)	1.28 (0.04)	−3.10	0.002	−0.15 (0.05)	−0.18 (0.05)			G_1 vs G_2	0.15 (−0.02, 0.32)	0.090
CRP (log_{10})	Per-protocol												
	MBSR+CCT (G_1)	39	-0.14 (0.13)	-0.08 (0.12)	0.38	0.708	0.06 (0.15)	-0.02 (0.11)	0.66	0.519			
	PE+CCT (G_2)	33	-0.05 (0.14)	-0.01 (0.13)	0.38	0.707	0.04 (0.11)	0.11 (0.12)					
	CCT−only (G_3)	34	-0.24 (0.12)	-0.11 (0.13)	0.94	0.346	0.13 (0.14)	0.15 (0.12)					
	Complete-case												
	MBSR+CCT (G_1)	43	−0.15 (0.13)	−0.13 (0.11)	0.15	0.882	0.02 (0.16)	−0.06 (0.11)	0.93	0.392			
	PE+CCT (G_2)	41	−0.10 (0.12)	−0.06 (0.12)	0.34	0.735	0.03 (0.10)	0.08 (0.11)					
	CCT−only (G_3)	39	−0.17 (0.11)	−0.08 (0.12)	0.71	0.477	0.09 (0.13)	0.14 (0.11)					

Note: This table reports the results of sensitivity analyses for VEGF and CRP using a lognormal distribution-based imputation method to estimate biomarker values below the detection limit. Non-detectable values were imputed using maximum likelihood estimation to fit a lognormal distribution to the observed data. After fitting the distribution, non-detectable values were imputed by randomly sampling from the portion of the fitted distribution below the lower limit of quantification. This approach ensures that imputed values reflect the natural distribution of the data while maintaining variability across samples. Five imputed datasets were generated, and all statistical analyses were conducted separately in each dataset. The final estimates were pooled using Rubin's rules. Abbreviations: CCT, computerized cognitive training; CRP, C-reactive protein; MBSR, Mindfulness-Based Stress Reduction; PE, physical exercise; Post-int, post-intervention; VEGF, vascular endothelial growth factor.

	Δ BDNF*	Δ IGF-1	Δ VEGF*	Δ CRP*	Δ IL-6*	Δ Cortisol
**Number of Strokes**	0.01	0.09	-0.01	0.12	0.00	0.07
**Type of Stroke**	0.18	0.02	0.02	-0.05	0.22	-0.31
**Type of Circulation**	-0.09	-0.09	-0.03	-0.08	0.04	0.06
**NIHSS Score**	-0.04	0.04	-0.08	0.03	0.09	0.01
**mRS Score**	-0.12	0.03	-0.06	-0.05	0.03	0.08
**Barthel Index**	0.11	0.02	0.10	-0.11	-0.07	-0.06
**MMSE Score**	-0.01	0.08	0.01	0.03	0.01	0.03
**BDI-II Score**	-0.12	-0.04	-0.17	-0.03	-0.16	-0.14

	Δ BDNF*	Δ IGF-1	Δ VEGF*	Δ CRP*	Δ IL-6*	Δ Cortisol
**Metabolic Syndrome**	0.08	0.06	0.05	0.16	0.11	0.06
**Antihypertensive Drugs**	0.07	-0.16	-0.01	0.18	0.00	-0.08
**Lipid-Lowering Drugs**	0.00	0.11	0.07	0.02	-0.07	0.05
**Antidiabetic Drugs**	0.15	-0.13	0.12	-0.17	-0.01	0.17
**Antidepressant Drugs**	-0.07	-0.01	-0.07	-0.03	-0.02	-0.15
**Anxiolytic Drugs**	0.02	0.06	-0.15	0.01	0.02	-0.12
**Smoking Status**	0.19	0.03	0.17	0.04	-0.06	-0.17
**Alcohol Intake**	0.06	0.02	0.09	0.20	0.05	0.06

	Δ BDNF*	Δ IGF-1	Δ VEGF*	Δ CRP*	Δ IL-6*	Δ Cortisol
**MEDAS Score**	0.01	-0.17	-0.04	0.04	0.04	0.11
**Δ MEDAS Score**	-0.08	0.12	-0.07	-0.11	-0.02	0.04
**P. Activity (METS)**	-0.01	-0.04	-0.01	-0.02	-0.06	0.06
**Δ P. Activity (METS)**	0.09	-0.20	0.16	0.23	0.03	0.06
**PSQI Score**	-0.10	0.00	-0.18	0.02	-0.07	-0.23
**Δ PSQI Score**	0.06	0.12	0.10	-0.23	-0.01	0.24
**Body Mass Index**	-0.12	0.01	-0.04	* 0.38	0.15	0.01
**Δ Body Mass Index**	0.09	0.03	0.07	-0.06	-0.04	-0.06

-1.0 -0.5 0.0 0.5 1.0

Figure S2. Partial correlation matrices of biomarker changes with clinical, medication, and lifestyle factors (per-protocol sample). This figure presents the Pearson partial correlation coefficients between changes (Δ) in blood biomarkers (BDNF, IGF-1, VEGF, CRP, IL-6, and cortisol) and various clinical, medication, and lifestyle variables, with correlations adjusted for age, sex, years of education, time since stroke, baseline biomarker concentration, and group assignment (modeled using two dummy-coded variables for the three groups). The categorical variables type of stroke (ischemic vs. hemorrhagic), circulation (anterior vs. posterior), metabolic syndrome (presence vs. absence), drug prescriptions (prescribed vs. not prescribed), and smoking status (current smoker vs. former/never smoker) were coded as dummy variables. Although presented in three panels, all 144 correlations underwent an FDR correction together, with only baseline BMI showing a significant positive correlation with changes in IL-6. Asterisks near biomarker names indicate log10-transformed values, while asterisks within the panels denote p-values after FDR correction (* $p_{adj} < 0.05$). Abbreviations: BDI-II, Beck Depression Inventory-II; BDNF, brain-derived neurotrophic factor; BMI, body mass index; CRP, C-reactive protein; FDR, false discovery rate; IGF-1, insulin-like growth factor 1; IL-6, interleukin 6; MEDAS, Mediterranean Diet Adherence Screener; METS, metabolic equivalent of task; MMSE, Mini-Mental State Examination; mRS, Modified Rankin Scale; NIHSS, National Institutes of Health Stroke Scale; PSQI, Pittsburgh Sleep Quality Index; VEGF, vascular endothelial growth factor.

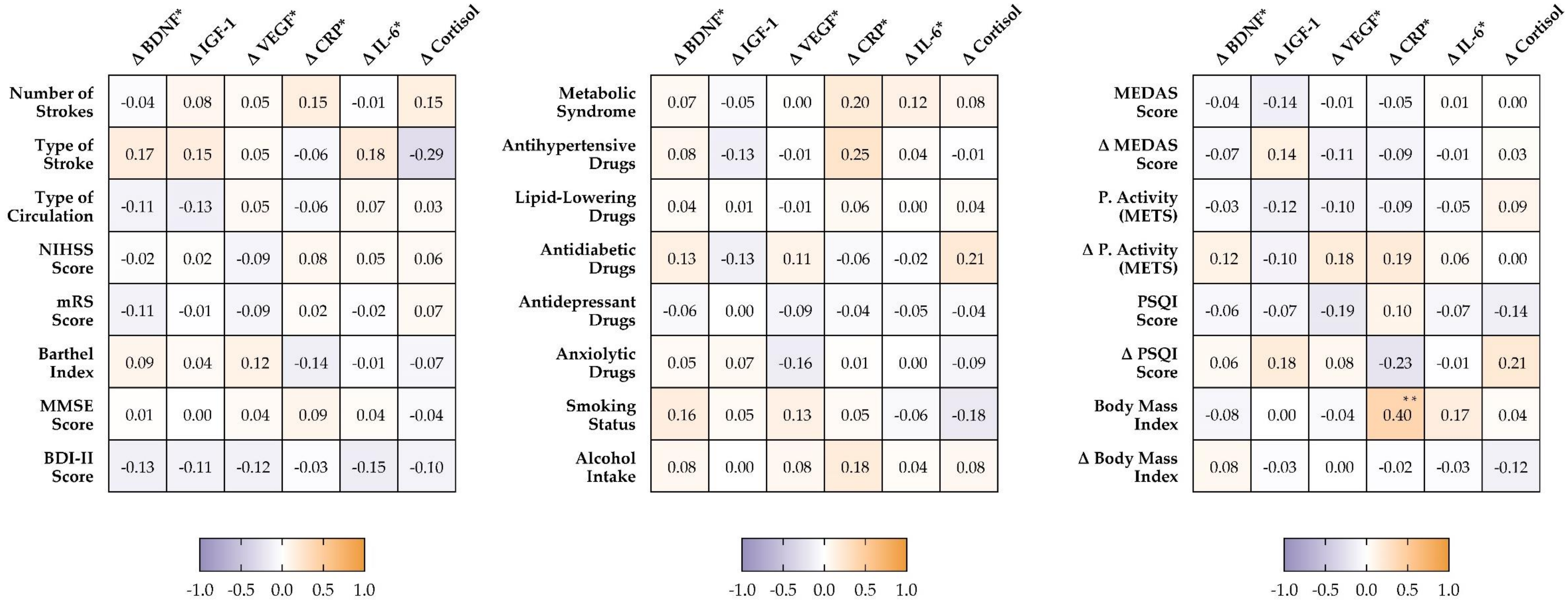

Figure S3. Partial correlation matrices of biomarker changes with clinical, medication, and lifestyle factors (complete-case sample). This figure presents the Pearson partial correlation coefficients between changes (Δ) in blood biomarkers (BDNF, IGF-1, VEGF, CRP, IL-6, and cortisol) and various clinical, medication, and lifestyle variables, with correlations adjusted for age, sex, years of education, time since stroke, baseline biomarker concentration, and group assignment (modeled using two dummy-coded variables for the three groups). The categorical variables type of stroke (ischemic vs. hemorrhagic), circulation (anterior vs. posterior), metabolic syndrome (presence vs. absence), drug prescriptions (prescribed vs. not prescribed), and smoking status (current smoker vs. former/never smoker) were coded as dummy variables. Although presented in three panels, all 144 correlations underwent an FDR correction together, with only baseline BMI showing a significant positive correlation with changes in IL-6. Asterisks near biomarker names indicate log10-transformed values, while asterisks within the panels denote p-values after FDR correction (** $p_{adj} < 0.01$). Abbreviations: BDI-II, Beck Depression Inventory-II; BDNF, brain-derived neurotrophic factor; BMI, body mass index; CRP, C-reactive protein; FDR, false discovery rate; IGF-1, insulin-like growth factor 1; IL-6, interleukin 6; MEDAS, Mediterranean Diet Adherence Screener; METS, metabolic equivalent of task; MMSE, Mini-Mental State Examination; mRS, Modified Rankin Scale; NIHSS, National Institutes of Health Stroke Scale; PSQI, Pittsburgh Sleep Quality Index; VEGF, vascular endothelial growth factor.

Table S8. Within-group changes in biomarker concentrations (complete-case sample)

Biomarker	Group	*n* *	Baseline (T_0) M (SD)	Post-int. (T_1) M (SD)	Difference score ($T_1 - T_0$) M (SD)	Difference score ($T_1 - T_0$) 95% CI †	*t*	*p*	p_{adj} †	*d*
**Growth factors**										
BDNF, pg/mL (log_{10})	MBSR+CCT	43	3.38 (0.60)	3.45 (0.56)	0.07 (0.40)	−0.08, 0.23	1.21	0.232	0.418	0.18
	PE+CCT	42	3.21 (0.60)	3.16 (0.45)	−0.05 (0.42)	−0.21, 0.11	−0.76	0.454	0.584	−0.12
	CCT-only	41	3.23 (0.43)	3.27 (0.42)	0.04 (0.31)	−0.08, 0.16	0.83	0.409	0.584	0.13
IGF-1, ng/mL	MBSR+CCT	43	98.79 (41.83)	84.76 (33.29)	−14.03 (23.24)	−22.87, −5.19	−3.96	**<0.001**	**0.003**	−0.60
	PE+CCT	42	89.46 (45.81)	74.42 (40.27)	−15.03 (26.01)	−25.05, −5.01	−3.74	**<0.001**	**0.003**	−0.58
	CCT-only	41	84.07 (37.32)	70.35 (34.54)	−13.72 (20.10)	−21.57, −5.88	−4.37	**<0.001**	**0.002**	−0.68
VEGF, pg/mL (log_{10})	MBSR+CCT	43	1.46 (0.34)	1.48 (0.39)	0.02 (0.47)	−0.16, 0.20	0.31	0.757	0.801	0.05
	PE+CCT	42	1.47 (0.38)	1.33 (0.30)	−0.14 (0.41)	−0.29, 0.02	−2.20	**0.034**	0.086	−0.34
	CCT-only	41	1.43 (0.30)	1.28 (0.27)	−0.16 (0.31)	−0.28, −0.04	−3.26	**0.002**	**0.008**	−0.51
**Inflammatory markers**										
CRP, pg/mL (log_{10})	MBSR+CCT	43	−0.12 (0.75)	−0.10 (0.65)	0.02 (0.91)	−0.32, 0.37	0.15	0.878	0.878	0.02
	PE+CCT	41	−0.08 (0.76)	−0.05 (0.72)	0.03 (0.53)	−0.18, 0.23	0.32	0.752	0.801	0.05
	CCT-only	39	−0.16 (0.62)	−0.07 (0.71)	0.09 (0.76)	−0.21, 0.40	0.77	0.445	0.584	0.12
IL-6, mg/L (log_{10})	MBSR+CCT	43	0.18 (0.40)	0.16 (0.36)	−0.02 (0.36)	−0.16, 0.12	−0.36	0.718	0.801	−0.06
	PE+CCT	42	0.23 (0.32)	0.26 (0.35)	0.03 (0.22)	−0.05, 0.12	1.02	0.314	0.513	0.16
	CCT-only	41	0.23 (0.34)	0.27 (0.29)	0.04 (0.22)	−0.04, 0.13	1.27	0.210	0.418	0.20
**Stress markers**										
Cortisol, ng/mL	MBSR+CCT	43	82.67 (25.05)	71.90 (21.30)	−10.77 (25.22)	−20.36, −1.18	−2.80	**0.008**	**0.023**	−0.43
	PE+CCT	42	85.13 (24.51)	74.41 (26.43)	−10.71 (19.01)	−18.03, −3.39	−3.65	**<0.001**	**0.003**	−0.56
	CCT-only	41	91.13 (24.83)	84.07 (25.19)	−7.07 (25.93)	−17.19, 3.05	−1.75	0.089	0.199	−0.27

Note: Unadjusted and adjusted *p*-values below 0.05 are in bold. * Statistics are based on the complete-case sample, including participants with data at both time points, regardless of intervention adherence. The sample size for CRP is slightly smaller because concentrations could not be quantified in three cases (two PE+CCT, one CCT-only) due to insufficient samples. † The 95% CI and adjusted *p*-values were FDR-corrected. Abbreviations: BDNF, brain-derived neurotrophic factor; CCT, computerized cognitive training; CRP, C-reactive protein; FDR, false discovery rate; IGF-1, insulin-like growth factor-1; IL-6, interleukin-6; MBSR, Mindfulness-Based Stress Reduction; PE, physical exercise; Post-int, post-intervention; VEGF, vascular endothelial growth factor.

Table S9. Mediation analyses of VEGF changes in relation to cognitive, mental health, mindfulness, and fitness outcomes (per-protocol and complete-case samples)

Dependent variable ($T_1 – T_0$)	Contrast	Per-protocol analysis	Complete-case analysis
		Coeff. (95% CI)	Coeff. (95% CI)
Verbal executive functioning	MBSR+CCT vs CCT-only	–0.04 (–0.13, 0.01)	–0.03 (–0.10, 0.02)
	PE+CCT vs CCT-only	0.00 (–0.04, 0.05)	0.00 (–0.04, 0.03)
	MBSR+CCT vs PE+CCT	–0.04 (–0.13, 0.01)	–0.02 (–0.10, 0.01)
Verbal memory	MBSR+CCT vs CCT-only	–0.02 (–0.14, 0.07)	0.01 (–0.10, 0.14)
	PE+CCT vs CCT-only	0.00 (–0.06, 0.03)	0.00 (–0.04, 0.08)
	MBSR+CCT vs PE+CCT	–0.02 (–0.12, 0.06)	0.01 (–0.08, 0.10)
Visual Executive Functioning	MBSR+CCT vs CCT-only	–0.02 (–0.13, 0.03)	0.01 (–0.09, 0.09)
	PE+CCT vs CCT-only	0.00 (–0.05, 0.02)	0.00 (–0.04, 0.03)
	MBSR+CCT vs PE+CCT	–0.02 (–0.11, 0.04)	0.01 (–0.09, 0.10)
Beck Depression Inventory-II	MBSR+CCT vs CCT-only	0.18 (–0.74, 1.24)	–0.29 (–1.84, 0.83)
	PE+CCT vs CCT-only	–0.06 (–0.70, 0.37)	0.03 (–0.62, 0.50)
	MBSR+CCT vs PE+CCT	0.25 (–0.93, 1.50)	–0.32 (–1.81, 0.95)
Depression Anxiety Stress Scale-21	MBSR+CCT vs CCT-only	1.01 (–1.53, 4.50)	0.20 (–3.63, 3.52)
	PE+CCT vs CCT-only	–0.41 (–3.07, 0.81)	–0.02 (–1.95, 0.97)
	MBSR+CCT vs PE+CCT	1.42 (–1.72, 5.89)	0.22 (–3.43, 4.26)
Mindful Attention Awareness Scale	MBSR+CCT vs CCT-only	–0.01 (–0.15, 0.12)	0.00 (–0.12, 0.12)
	PE+CCT vs CCT-only	0.00 (–0.05, 0.07)	0.00 (–0.04, 0.05)
	MBSR+CCT vs PE+CCT	–0.01 (–0.17, 0.14)	0.00 (–0.13, 0.13)
Five Facet Mindfulness Questionnaire	MBSR+CCT vs CCT-only	0.01 (–0.05, 0.09)	0.03 (–0.03, 0.12)
	PE+CCT vs CCT-only	0.00 (–0.04, 0.03)	0.00 (–0.04, 0.04)
	MBSR+CCT vs PE+CCT	0.02 (–0.06, 0.10)	0.03 (–0.03, 0.13)
30-second Chair Stand Test	MBSR+CCT vs CCT-only	–0.07 (–0.47, 0.17)	–0.10 (–0.46, 0.12)
	PE+CCT vs CCT-only	0.01 (–0.13, 0.18)	–0.01 (–0.19, 0.12)
	MBSR+CCT vs PE+CCT	–0.09 (–0.50, 0.20)	–0.09 (–0.42, 0.13)
30-second Arm Curl Test	MBSR+CCT vs CCT-only	–0.09 (–0.54, 0.21)	–0.16 (–0.59, 0.12)
	PE+CCT vs CCT-only	0.01 (–0.11, 0.25)	–0.02 (–0.22, 0.18)
	MBSR+CCT vs PE+CCT	–0.11 (–0.62, 0.18)	–0.14 (–0.56, 0.10)
2-minute Step Test	MBSR+CCT vs CCT-only	–0.88 (–3.51, 0.78)	–0.48 (–2.69, 1.28)
	PE+CCT vs CCT-only	0.17 (–0.82, 1.52)	–0.03 (–0.85, 0.85)
	MBSR+CCT vs PE+CCT	–1.05 (–3.95, 0.82)	–0.45 (–2.66, 1.01)

Note: This table presents the results of mediation analyses assessing whether changes in VEGF mediated the effects of the interventions on cognitive, mental health, mindfulness, and physical fitness outcomes. The dependent variables represent the pre-to-post-intervention change scores. The coefficients correspond to the indirect effects of each intervention contrast (MBSR+CCT vs. CCT-only, PE+CCT vs. CCT-only, and MBSR+CCT vs. PE+CCT) through VEGF. Mediation models were estimated using the PROCESS macro in R with bias-corrected bootstrap resampling (10,000 iterations) to compute confidence intervals. The analysis was conducted separately for the per-protocol sample (participants with ≥80% adherence) and the complete-case sample (participants with available biomarker data at both time points). A mediation effect was considered significant if the 95% Bonferroni-corrected confidence interval did not include zero. No mediation effects reached statistical significance. *Abbreviations:* CCT, computerized cognitive training; MBSR, Mindfulness-Based Stress Reduction; PE, physical exercise; VEGF, vascular endothelial growth factor.

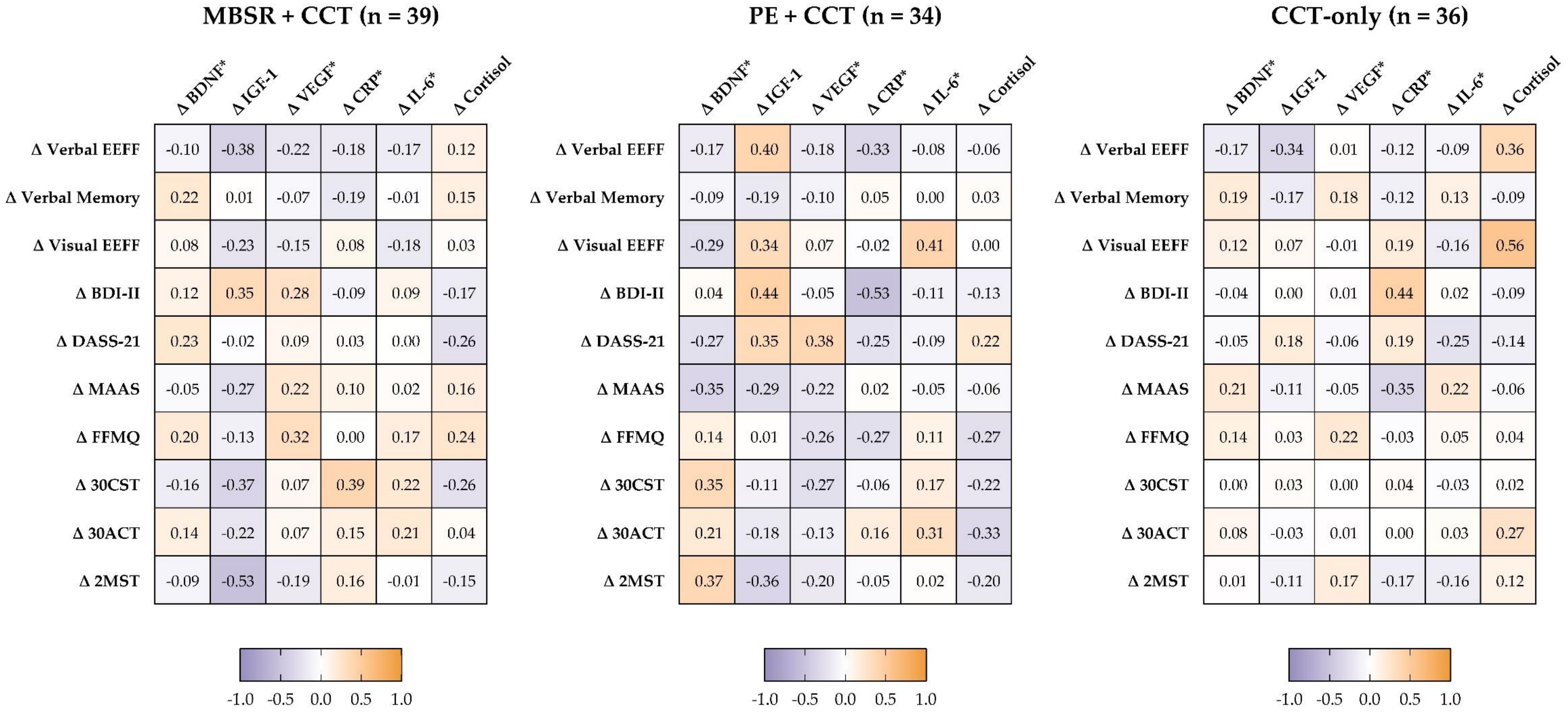

Figure S4. Partial correlation matrices of biomarker changes with behavioral outcomes stratified by intervention group (per-protocol sample). This figure presents Pearson partial correlation coefficients between changes (Δ) in blood biomarkers (BDNF, IGF-1, VEGF, CRP, IL-6, and cortisol) and behavioral outcomes (cognitive, mental health, mindfulness, and fitness outcomes) stratified by intervention group (MBSR+CCT, PE+CCT, and CCT-only). Correlations were adjusted for age, sex, years of education, time since stroke, and baseline biomarker concentration. FDR correction was applied independently within each group, and no correlations survived FDR correction in any group. Asterisks near biomarker names indicate log10-transformed values. Abbreviations: 30ACT, 30-second Arm Curl Test; 30CST, 30-second Chair Stand Test; 2MST, 2-Minute Step Test; BDI-II, Beck Depression Inventory-II; BDNF, brain-derived neurotrophic factor; CRP, C-reactive protein; DASS-21, Depression Anxiety Stress Scales-21; EEFF, executive functioning; FDR, false discovery rate; FFMQ, Five Facet Mindfulness Questionnaire; IGF-1, insulin-like growth factor 1; IL-6, interleukin 6; MAAS, Mindful Attention Awareness Scale; VEGF, vascular endothelial growth factor.

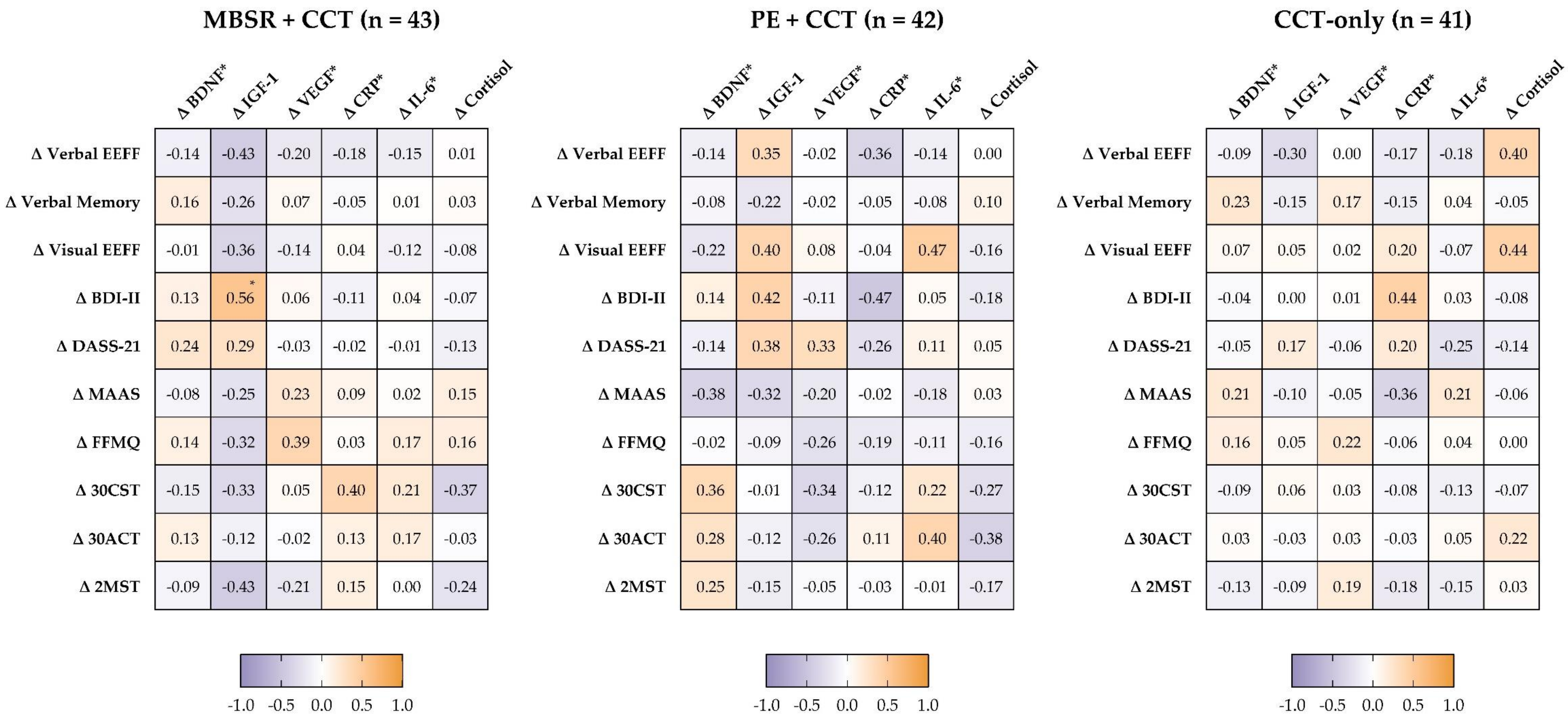

Figure S5. Partial correlation matrices of biomarker changes with behavioral outcomes stratified by intervention group (complete-case sample). This figure presents Pearson partial correlation coefficients between changes (Δ) in blood biomarkers (BDNF, IGF-1, VEGF, CRP, IL-6, and cortisol) and behavioral outcomes (cognitive, mental health, mindfulness, and fitness outcomes) stratified by intervention group (MBSR+CCT, PE+CCT, and CCT-only). Correlations were adjusted for age, sex, years of education, time since stroke, and baseline biomarker concentration. FDR correction was applied independently within each group, and the only significant correlation was a positive one between IGF-1 and BDI-II in the CCT-only group (* $p_{adj} < 0.05$). Asterisks near biomarker names indicate log10-transformed values. Abbreviations: 30ACT, 30-second Arm Curl Test; 30CST, 30-second Chair Stand Test; 2MST, 2-Minute Step Test; BDI-II, Beck Depression Inventory-II; BDNF, brain-derived neurotrophic factor; CRP, C-reactive protein; DASS-21, Depression Anxiety Stress Scales-21; EEFF, executive functioning; FDR, false discovery rate; FFMQ, Five Facet Mindfulness Questionnaire; IGF-1, insulin-like growth factor 1; IL-6, interleukin 6; MAAS, Mindful Attention Awareness Scale; VEGF, vascular endothelial growth factor.
